# Serum vesicle biomarkers reflect the disease activity of idiopathic pulmonary fibrosis

**DOI:** 10.1186/s12967-025-07011-2

**Published:** 2025-10-15

**Authors:** Yuya Shirai, Takatoshi Enomoto, Yoshito Takeda, Ryuya Edahiro, Miho Takahashi-Itoh, Yoshimi Noda, Yuichi Adachi, Mana Nakayama, Takahiro Kawasaki, Taro Koba, Yu Futami, Hanako Yoshimura, Saori Amiya, Reina Hara, Makoto Yamamoto, Daisuke Nakatsubo, Yasuhiko Suga, Maiko Naito, Kentaro Masuhiro, Takanori Matsuki, Haruhiko Hirata, Kota Iwahori, Izumi Nagatomo, Kotaro Miyake, Shohei Koyama, Kiyoharu Fukushima, Takayuki Shiroyama, Yujiro Naito, Shinji Futami, Yayoi Natsume-Kitatani, Naoko Ose, Soichiro Funaki, Satoshi Nojima, Shigeyuki Shichino, Masahiro Yanagawa, Yasushi Shintani, Mari Nogami-Itoh, Jun Adachi, Yoshikazu Inoue, Takeshi Tomonaga, Yukinori Okada, Kenji Mizuguchi, Atsushi Kumanogoh

**Affiliations:** 1 Department of Respiratory Medicine and Clinical Immunology, Graduate School of Medicine, The University of Osaka, 2-2 Yamadaoka, Suita, Osaka 565-0871 Japan; 2 Department of Statistical Genetics, Graduate School of Medicine, The University of Osaka, Suita, Osaka Japan; 3 Laboratory of Statistical Immunology, Immunology Frontier Research Center (WPI-IFReC), The University of Osaka, Suita, Osaka Japan; 4 Department of Respiratory Medicine, Kinki Central Hospital of the Mutual Aid Association of Public School Teachers, Itami, Hyogo Japan; 5 Department of Respiratory Medicine, National Hospital Organization Osaka Toneyama Medical Center, Toyonaka, Osaka Japan; 6 Laboratory of Bioinformatics, Artificial Intelligence Center for Health and Biomedical Research, National Institutes of Biomedical Innovation, Health and Nutrition, 7-6-8 Saito-Asagi, Ibaraki, Osaka Japan; 7 Institute of Advanced Medical Sciences, Tokushima University, Tokushima, Japan; 8 Department of General Thoracic Surgery, Graduate School of Medicine, The University of Osaka, Suita, Osaka Japan; 9 Department of Pathology, Graduate School of Medicine, The University of Osaka, Suita, Osaka Japan; 10 Division of Molecular Regulation of Inflammatory and Immune Diseases, Research Institute of Biomedical Sciences, Tokyo University of Science, Chiba, Japan; 11 Department of Radiology, Graduate School of Medicine, The University of Osaka, Suita, Osaka Japan; 12 Laboratory of Proteomics for Drug Discovery, Center for Drug Design Research, National Institutes of Biomedical Innovation, Health and Nutrition, Osaka, Japan; 13 Clinical Research Center, National Hospital Organization Kinki-Chuo Chest Medical Center, Osaka, Japan; 14 Laboratory for Systems Genetics, RIKEN Center for Integrative Medical Sciences, Yokohama, Japan; 15 Department of Genome Informatics, Graduate School of Medicine, The University of Tokyo, Tokyo, Japan; 16 Center for Infectious Diseases for Education and Research (CiDER), The University of Osaka, Suita, Osaka Japan; 17 Integrated Frontier Research for Medical Science Division, Institute for Open and Transdisciplinary Research Initiatives (OTRI), The University of Osaka, Suita, Osaka Japan; 18 Laboratory of In Silico Design, Artificial Intelligence Center for Health and Biomedical Research, National Institutes of Biomedical Innovation, Health and Nutrition, 7-6-8 Saito-Asagi, Ibaraki, Osaka Japan; 19 Institute for Protein Research, The University of Osaka, 3-2 Yamadaoka, Suita, Osaka Japan; 20 Department of Immunopathology, Immunology Frontier Research Center (WPI-IFReC), The University of Osaka, Suita, Osaka Japan; 21 Japan Agency for Medical Research and Development–Core Research for Evolutional Science and Technology (AMED–CREST), The University of Osaka, Osaka, Japan; 22 Center for Advanced Modalities and DDS (CAMaD), The University of Osaka, Suita, Japan

**Keywords:** Idiopathic pulmonary fibrosis, Non-target proteomics, Extracellular vesicles, SFTPB

## Abstract

**Background:**

Idiopathic pulmonary fibrosis (IPF) is a heterogeneous disease caused by an interplay of genetic and environmental factors. Biomarkers that reflect the progression of fibrosis are required for the management of IPF.

**Methods:**

We extracted serum extracellular vesicles from a discovery cohort (127 IPF patients and 34 controls) and a validation cohort (20 IPF patients and 22 controls). Non-targeted proteomic analysis was performed by a data-independent acquisition method. We investigated the proteomic profiles in relation to multiple clinical parameters associated with IPF. To further evaluate the biological relevance of the identified biomarkers, we analyzed publicly available single-cell RNA sequencing datasets of lung tissue and conducted immunochemical validation using our collected lung samples.

**Results:**

We obtained 2420 protein profiles in serum extracellular vesicles and identified 19 IPF-associated proteins; their expressions were significantly lung-specific. Protein module analyses revealed that the upstream components of the complement system were increased in IPF. These IPF-associated proteins were involved in various IPF-associated genes and heterogeneously increased in IPF patients. Notably, surfactant protein B (SFTPB) not only showed superior diagnostic performance over the existing marker but was also significantly associated with progressive disease activity, such as the extent of fibrosis and decline in lung function. Furthermore, single-cell RNA-sequencing analysis revealed that SFTPB was associated with the TGF-β/SMAD pathway in *SCGB3A2* + cells in IPF lungs. SFTPB expression in *SCGB3A2* + cells was confirmed by immunostaining.

**Conclusions:**

Serum extracellular vesicles could capture heterogenetic fibrotic profiles in IPF, and SFTPB can be a promising biomarker reflecting the disease activity.

**Supplementary Information:**

The online version contains supplementary material available at 10.1186/s12967-025-07011-2.

## Introduction

Idiopathic pulmonary fibrosis (IPF) is an intractable disease with irreversible fibrosis of the lungs, characterized by progressive worsening of dyspnoea and lung function, leading to a poor prognosis [1]. The clinical course of IPF varies depending on the patient, with some patients remaining stable for many years and others developing progressive fibrosis. This diversity in the clinical course arises from the differences in the pathophysiology of IPF [2]. Therefore, novel biomarkers that reflect the individual endotypes and disease activity in heterogeneous IPF etiologies are warranted.

Peripheral blood is an ideal resource of biomarkers that can be obtained repeatedly in routine clinical practice. However, approximately 22 proteins, including albumin and immunoglobulins, account for 99% of all serum proteins, and these serum contaminants mask small amounts of biomarker candidates when measured using conventional liquid chromatography-mass spectrometry (LC–MS/MS) [3]. To overcome this obstacle, we focused on serum extracellular vesicles (EVs) because they contain fewer serum contaminants and prevent proteolysis by serum proteases [4]. EVs have been increasingly acknowledged as important carriers of biological cargo that play key roles in intercellular communication by transferring protein content, such as cytokines and growth factors [5]. Thus, EV constituents play different pathological roles in various diseases, including malignancies, inflammatory diseases, and infection [6, 7]. The association between plasma EVs and IPF in microRNAs has been studied [8]. A previous study reported that EVs in bronchoalveolar lavage fluid functioned as carriers for signaling mediators, such as WNT5A [9]. However, the association between the clinical profile of IPF and proteins in serum EVs remains elusive, hindering their clinical application as useful biomarkers reflecting disease activity.

Here, we report the results of a proteomic analysis of serum EVs in IPF patients and healthy controls. We utilized the latest proteomics technology, data-independent acquisition (DIA), to comprehensively capture the protein profiles in serum EVs. The identified IPF-associated proteins were evaluated for their association with multiple clinical features of IPF. Moreover, we assessed the biological significance of surfactant protein B (SFTPB), a promising biomarker for disease activity, using single-cell RNA-sequencing (scRNA-seq) analysis of IPF lungs.

## Methods

### Study participants and sample collection

This study enrolled 127 IPF cases and 34 healthy controls collected from the Osaka University Hospital. IPF was diagnosed through multidisciplinary discussion (MDD) based on the American Thoracic Society and European Respiratory Society guidelines [10]. We excluded the participants with acute exacerbations or active infections. We collected 10 mL of blood from each participant, centrifuged at 3000 rpm for 10 min, and the supernatant was separated as serum. The serum was immediately frozen and stored in a − 80 °C freezer.

### Protocol for proteomic analysis

EV isolation was performed by an affinity capture isolation method [11]. Briefly, phosphatidylserine-positive EVs were purified from 100 μL of serum using the MagCapture isolation kit (Fujifilm Wako). The presence of isolated EVs was confirmed using transmission electron microscopy. Size distributions and numbers were confirmed by nanoparticle tracking analysis, NanoSight [12]. We evaluated the number and size of EVs between the cases and controls using Wilcoxon rank-sum test. The proteins in EVs were reduced with tris (2-carboxyethyl) phosphine, alkylated with iodoacetamide, trypsin digested, and desalted. The pretreated samples were subjected to LC–MS/MS analysis using DIA analysis software, Spectronaut, and run-wise imputation was performed for missing values. One commercial serum sample was added to every 15 samples as a quality control to assure quality from sample preparation to data analysis. DIA analysis of digested HeLa cells was also performed as a quality control for mass spectrometry. The proteomics abundance data were normalized using variance stabilization normalization (VSN) method [13] implemented in limma R package.

### Case–control association test for individual protein abundance

We performed association tests for the individual normalized protein abundance using lm function in the R (version 3.6.1) and evaluated the effect size of the disease state. We designed our regression model with IPF or not as the explanatory variable and normalized protein abundance as the response variable. Age and sex were included as covariates in the regression model. We set the threshold for significant enrichment as *P* = 0.05/2420, adjusted by Bonferroni correction. We performed a hypergeometric test of the biological pathway for significant IPF-associated proteins using ReactomePA [14] and Clusterprofiler [15].

### Replication analysis for IPF-associated proteins

We evaluated the associations in an independent cohort for 19 IPF-associated proteins identified in the discovery cohort. The replication cohort was composed of 20 IPF cases and 23 healthy controls. Participant enrolment, sample preparation, and association tests were performed in the same manner as the discovery cohort.

### Receiver-operating characteristic (ROC) analysis

In this analysis, we selected 121 IPF cases and 13 controls with no missing data on serum KL6 and proteomics in the discovery cohort. We evaluated the performance of a classifier between IPF and control using pROC R package [16]. We used the Youden index to find the optimal cutoff value for sensitivity and specificity.

### Enzyme-linked immunosorbent assay

We quantified the serum SFTPB amounts for the same serum specimens from which we extracted EVs in the replication cohort, using the SEB622Hu ELISA kit (Cloud-Clone Corp., Houston, TX, USA) according to the manufacturer’s instructions.

### Tissue expression assessment of proteins in serum EVs

We referred to the RNA consensus tissue gene data of the Human Protein Atlas [17] to investigate the tissue expression profiles of proteins in serum EVs. To compare the tissue expression profiles among proteins, the expression levels in each tissue were divided by the maximum value and scaled between 0 and 1. We visualized the scaled expression levels in a circular plot for all proteins in serum EVs using Circlize R package [18] and a heat map for the IPF-associated proteins in the discovery cohort using ComplexHeatmap R package [19]. As a metric of tissue specificity, we calculated the Tau score for each protein and defined a tissue-specific protein as one with a score greater than 0.8 [20]. We defined tissue-specific proteins with maximal expression in lung tissue as lung-specific proteins. We applied Fisher's exact test to compare the difference in the distribution of the Tau scores.

### Case−control association test and enrichment analysis for protein modules

We used weighted correlation network analysis (WGCNA) algorithm for protein module analysis, as previously described [21]. A weighted protein co-expression network was generated using the 2420 normalized protein abundance × 161 sample matrix. We calculated the adjacency with a “signed network” option and soft threshold power of the adjacency matrix set to 5, created Topological Overlap Matrix using TOMsimilarity, calculated the gene tree using hclust against 1-TOM with method = “average”, and conducted a dynamic tree cut with the following parameters: deepSplit = 2 and minClusterSize = 25. Module eigenproteins (MEs) were calculated using the moduleEigengenes function. As in the association test for the individual protein, we evaluated the effect size of disease state for each ME in the linear regression model, including age and sex as covariates.

We performed a hypergeometric test for IPF-associated module using ReactomePA. We also performed gene set enrichment analysis (GSEA) [22] of the Reactome pathway with gsePathway function, using the t statistics obtained from the case–control association test of individual protein as input.

### Association test for clinical features of IPF

In this analysis, we selected 121 IPF cases and 13 controls with no missing data on serum KL6 and proteomics in the discovery cohort. Since VSN normalization generalized the logarithmic function of base 2 (glog2) to proteomics data, we applied the glog2 function of the Mkmisc R package to serum KL6, and then standardised the IPF-associated proteins and serum KL6 to z-scores to compare under the same conditions. We designed our regression model with each IPF feature as the explanatory variable and normalized protein abundance as the response variable, including age and sex as covariates. We evaluated the effect size of the following IPF features: the extent of fibrosis on CT, percent-predicted forced vital capacity (%FVC), percent-predicted diffusing capacity of the lung for carbon monoxide progressive phenotype (%DLCO), progressive phenotype, and the complication of lung emphysema or lung cancer. The extent of fibrosis on CT was binned into < 10%, 10–50%, or 50% < depending on the ratio to the total lung. Progressive phenotype defined using the criteria of the INBUILD trial [23]. Briefly, progressive phenotype patients met the following criteria within two years before or after the time of blood collection: a relative decline in FVC of at least 10% of the predicted value, a relative decline in FVC of 5% to less than 10% of the predicted value, and worsening of respiratory symptoms or an increased extent of fibrosis on CT, or worsening of respiratory symptoms and an increased extent of fibrosis.

#### Network analysis integrating IPF-associated proteins in EVs and known IPF-associated genes

We queried the 19 IPF-associated proteins, one IPF-associated protein module, and known IPF-associated genes [1, 24, 25] in STRING V.11.5 [26], a database that collected protein–protein interaction (PPI) networks. In STRING, each PPI is annotated with a score between 0 and 1 based on the physical and functional information. We drew a network diagram for the relationships with a score of 0.5 or higher using Cytoscape [27].

#### Western blotting

To examine the SFTPB amount in various respiratory diseases, serum EVs were newly extracted from the following participants: healthy controls (n = 6), IPF (n = 9), sarcoidosis (n = 5), mycobacterium avium complex (n = 5), chronic hypersensitivity pneumonitis (n = 5), lung cancer (n = 7), chronic obstructive pulmonary disease (n = 4), and bronchial asthma patients (n = 4).

Protein samples were loaded onto NuPAGE 4–12% Bis–Tris gels (Invitrogen). For immunoblot analysis, the gels were electroblotted onto polyvinylidene difluoride membranes (Bio-Rad). Membranes were blocked with Blocking One (Nacalai Tesque) at room temperature for 60 min, incubated with the specific primary antibody, and then incubated with the appropriate secondary antibody. The following primary antibodies were used for immunoblotting. Primary antibody: mouse anti-human SFTPB (sc-133143; Santa Cruz Biotechnnology) diluted to 1/100 in can get signal solution 1 (TOYOBO) at room temperature for 120 min. Secondary antibody: sheep anti-mouse IgG (NA931v; Cytiva) diluted to 1/5000 in can get signal solution 2 (TOYOBO) at room temperature for 60 min. The immunoreactive signals were visualized using SuperSignal West Atto Ultimate Sensitivity Maximum Chemiluminescent Substrate (Thermo Fisher Scientific) and detected on a ChemiDoc Touch (Bio-Rad). ImageJ [28] was used to obtain densitometry and the Wilcoxon rank-sum tests were performed between healthy controls and individual diseases.

#### SFTPB expression analysis for lung single-cell RNA-sequencing (scRNA-seq) data

We analyzed the lung scRNA-seq data provided by Habermann et al. [29] consisting of 12 IPF cases and 10 controls. This dataset was prepared by extracting IPF cases and controls from the data originally generated from the peripheral lung tissue of explanted lungs from 20 patients with pulmonary fibrosis and 10 nonfibrotic controls (declined donors). To clarify the cell type-specific *SFTPB* expression, we created the density plot using the plot_density function of the Nebulosa R package [30]. To examine the primary cell sources of *SFTPB* expression in IPF lungs, we transformed *SFTPB* raw count data into count per million (CPM) by normalizing with a scale size factor of 1,000,000 and estimated the summed CPM amount per each cell type as pseudobulk data. Furthermore, we performed differential expression (DE) analysis between IPF and controls. In the DE analysis, the pseudobulk data were first prepared by aggregating the *SFTPB* raw counts for each cell type within each sample. We applied a negative binomial test to the pseudo-bulk data using the Bioconductor package edgeR [31].

#### Differential abundance analysis

We applied Milo [32] to evaluate the differential abundance of cells between IPF and control lungs within defined neighborhoods. We first used the buildGraph function to construct a *k*-nearest neighbor (KNN) graph with k = 30, using 30 principal components (d = 30). We subsequently used the makeNhoods function to assign the cells to neighborhoods based on their connectivity over the KNN graph. To test for differential abundance, Milo fit a negative binomial generalized linear model to the counts for each neighborhood, accounting for different numbers of cells across samples using TMM normalization. To control for multiple testing, we adapted the spatial FDR implemented in Milo. The spatial FDR and log2 fold change of the number of cells between the two conditions in each neighborhood were used for visualization.

#### SFTPB co-expression analysis and enrichment analysis

We identified *SFTPB* co-expression genes in type 2 alveolar epithelial (AT2) and *SCGB3A2* + cells in IPF lung using the Bioconductor hdWGCNA package, that is WGCNA algorithm optimized for single-cell sparse data [33]. We used the ConstructNetwork function with soft threshold power of the adjacency matrix set to 9. We performed a hypergeometric test for the *SFTPB* co-expressed genes using ReactomePA.

#### Validation analysis for SFTPB expressing cells

To verify the changes in *SFTPB* expressing cells, we prepared a validation dataset by excluding scRNA-seq data by Habermann et al. [29] from the dataset in Human Lung Cell Atlas [34]. As a result, this validation dataset was composed of 55 IPF and 52 controls from three datasets [35–37]. We projected the cell types from the original data to the validation data using a spatial data structure (cKDTree implemented in scirpy) to efficiently find the nearest neighbors between them. We summed the normalized expression levels for each projected cell type, analogous to the original pseudobulk analysis. Because raw count data was not accessible in the validation dataset, we evaluated SFTPB expression changes between IPF and HC lungs using Wilcoxon rank-sum test instead of edgeR employing the negative binomial model.

#### Immunohistochemistry

IPF tissue samples were obtained from IPF patients who underwent surgery for suspected lung cancer. We confirmed SFTPB expression via DAB staining and co-expression of SFTPB and SCGB3A2 via fluorescent staining by the following procedure.

#### DAB staining

Paraffin-fixed tissues were deparaffinized using xylene and alcohol, incubated with 10 mmol/L citrate buffer (pH 6.0) for antigen retrieval, oxidized using 3% hydrogen peroxide at room temperature for 10 min, and blocked with 3% bovine serum albumin in phosphate-buffered saline at room temperature for 1 h. The slides were incubated with anti-SFTPB (1:200) (sc-133143; Santa Cruz Biotechnology), followed by incubation with horseradish peroxidase-conjugated anti-mouse (414132F; Nichirei Biosciences) at room temperature for 60 min. Image acquisition was performed with an OLYMPUS-BX51 microscope.

#### Fluorescent staining

The paraffin-fixed tissues were deparaffinized using xylene and alcohol, incubated with Target Retriever Solution, pH 9.0 (S2368; Dako) for antigen retrieval, and blocked with Blocking One Histo (06349-64; Nacalai Tesque) at room temperature for 30 min. The slides were incubated with anti-SFTPB (1:30) (sc-133143; Santa Cruz Biotechnology) and anti-SCGB3A2 antibody (1:30) (26,228-1-AP; Proteintech) at room temperature for 2 h, followed by incubation with goat anti-mouse IgG Alexa 488 (1:250) (A-11017; Invitrogen) and goat anti-rabbit IgG Alexa 594 (1:250) (A-11072; Invitrogen) at room temperature for 60 min. Image acquisition was performed with a Keyence BZ-X700 fluorescence microscope.

## Results

### Associations between serum EVs proteins and IPF

**Fig. 1:**
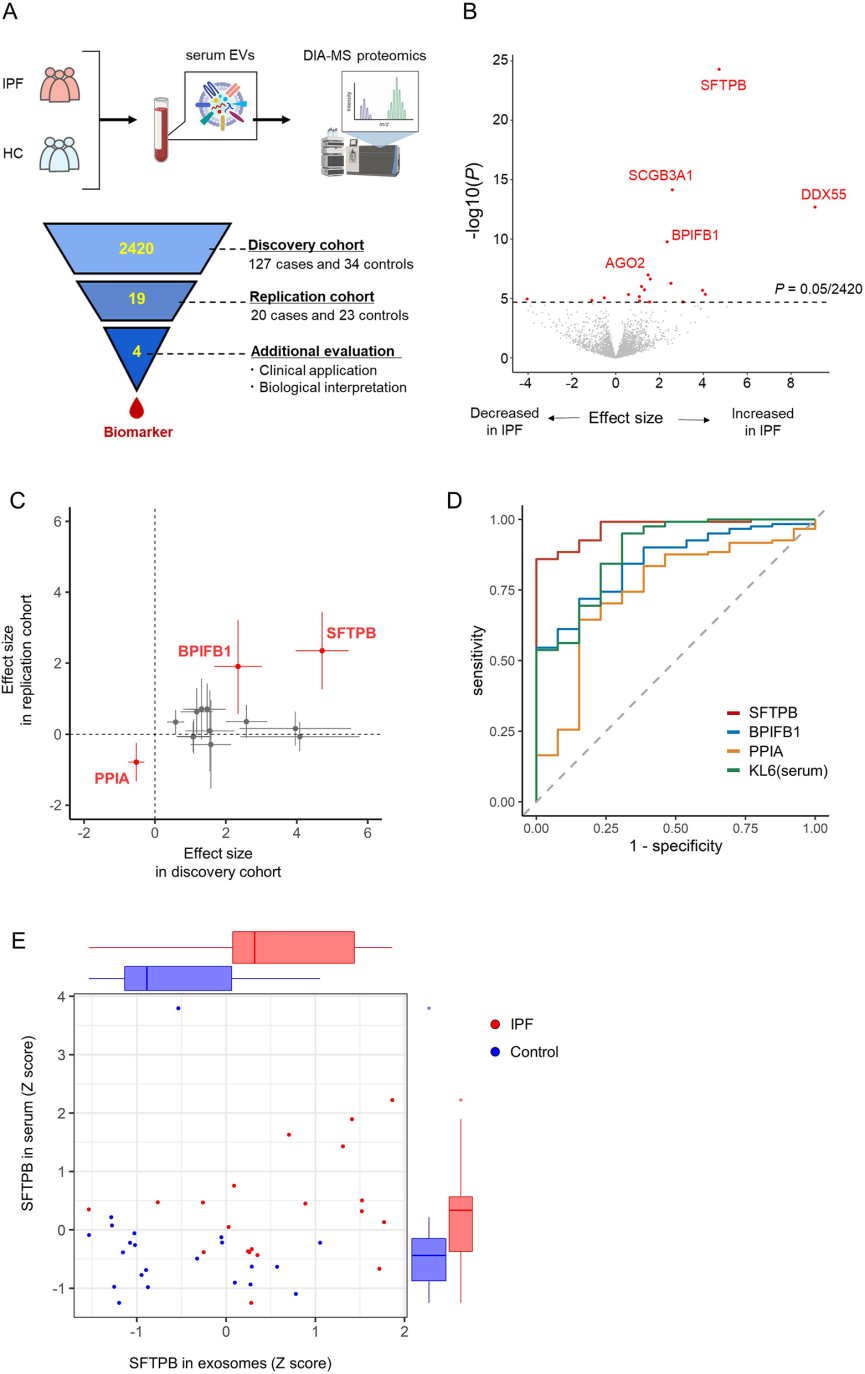
Study overview and association analysis of proteome in serum EVs from IPF patients and controls. **A** Overview of the study design. The data-independent acquisition mass spectrometric (DIA-MS) analysis was applied to serum EVs obtained from IPF patients and healthy controls. The output data were assessed through case–control association tests, and IPF-associated proteins were examined for clinical utility and biological significance. The figure was created using BioRender.com. **B** A volcano plot of the association test in the discovery cohort. The x-axis indicates effect sizes in linear regression. The y-axis indicates − log10(*P*) of association of each protein. The horizontal dashed line indicates the Bonferroni-corrected threshold. Significant proteins above the threshold are coloured in red. Top five proteins significantly associated proteins are shown. **C** A scatter plot describing the effect sizes of the discovery (x-axis) and replication (y-axis) cohort. The dots indicate the effect size of each protein, and the whiskers represent 95% confidence intervals. Proteins associated significantly in both cohorts are highlighted in red. **D** ROC curves of three significant proteins in both cohorts and serum KL6. **E** A scatter plot describing scaled SFTPB in EVs quantified by DIA-MS and in serum quantified by ELISA. The box plots represent the marginal distributions. EVs, extracellular vesicles; IPF, idiopathic pulmonary fibrosis; DIA-MS, data-independent acquisition mass spectrometric spectrometry; ROC, receiver operating characteristic

We identified as many as 2420 proteins in EVs from < 50 µL of serum from 127 IPF patients and 34 healthy controls through LC–MS/MS analysis using the DIA method (Fig. 1A). Table E1 provides a summary of the participants. The EVs isolated from IPF cases and controls similarly expressed a general EV marker protein, CD9, on their surfaces (Fig. E1A), and the number and size were comparable between them (*P* = 0.44; Fig. E1B–E). All 2420 proteins were assessed by association tests between IPF cases and controls. We found 19 significantly IPF-associated proteins; the expressions of 16 were increased, and those of three were decreased in IPF patients (Fig. 1B, Table 1). Four of the 19 significant proteins were previously associated with IPF: SCGB3A1, TGFBI, SFTPA, and CCL18 [38, 39]. The IPF cases and controls were separated in the principal component analysis of the 19 IPF-associated proteins (Fig. E2). SFTPB showed the most significant association with IPF (effect size = 4.72, *P* = 5.0 × 10^–25^). Pathway enrichment analysis showed that these 19 IPF-associated proteins were significantly enriched in the surfactant metabolism (false discovery rate, FDR = 0.0034) and cell adhesion molecule binding (FDR = 0.047) (Fig. E3). In the replication analysis, 16/19 proteins were successfully quantified. Three of these proteins were nominally significant (*P* < 0.05) and consistent with the direction of effect in the discovery cohort (SFTPB, effect size = 2.35, *P* = 1.4 × 10^–4^, BPI Fold Containing Family B Member 1 (BPIFB1), effect size = 1.90, *P* = 0.0075; PPIA, effect size = − 0.79, *P* = 0.0074; Fig. 1C). For clinical application, we compared the diagnostic performance of these three biomarker candidates with that of serum KL6, a representative IPF-related marker. ROC curve analysis showed that the areas under the curve (AUROC) of SFTPB, BPIFB1, PPIA, and serum KL6 levels were 0.97, 0.85, 0.75, and 0.89, respectively (Fig. 1D, Table E2). Notably, the SFTPB specificity was 1.00 and was much better than the KL6 specificity of 0.69. Thus, SFTPB was superior to serum KL6, and BPIFB1 was comparable to serum KL6 in IPF diagnosis.

**Table 1:** Summary of the 19 IPF-associated proteins in serum EVs

UniProtKB/Swiss-Prot	Symbol	Description	FC*	Effect size	SE	*P*
P07988	SFTPB	Surfactant protein B	13.9	4.72	0.38	5.0 × 10^–25^
Q96QR1	SCGB3A1	Secretoglobin family 3A member 1	5.7	2.58	0.30	7.0 × 10^–15^
Q8NHQ9	DDX55	DEAD-Box helicase 55	1.3	9.09	1.13	1.9 × 10^–13^
Q8TDL5	BPIFB1	BPI fold containing family B member 1	5.0	2.34	0.34	1.7 × 10^–10^
Q9UKV8	AGO2	Argonaute RISC catalytic component 2	3.3	1.47	0.26	1.0 × 10^–7^
Q9UGI8	TES	Testin LIM domain protein	1.9	1.58	0.29	2.3 × 10^–7^
Q96RF0	SNX18	Sorting Nexin 18	4.3	2.52	0.48	5.3 × 10^–7^
Q15582	TGFBI	Transforming growth factor Beta induced	1.8	1.18	0.23	9.6 × 10^–7^
Q8IWL1; Q8IWL2	SFTPA1; SFTPA2	Surfactant protein A1; A2	2.3	1.31	0.27	1.8 × 10^–6^
Q5BJH7	YIF1B	Yip1 interacting factor Homolog B, membrane trafficking protein	8.0	3.96	0.80	2.0 × 10^–6^
P0DP57; P0DP58-2	SLURP2; LYNX1	Secreted LY6/PLAUR domain containing 2; Ly6/Neurotoxin 1	2.9	4.08	0.86	4.4 × 10^–6^
Q96FN4	CPNE2	Copine 2	1.4	0.58	0.12	4.6 × 10^–6^
P55774	CCL18	C–C Motif Chemokine Ligand 18	2.1	1.08	0.23	7.0 × 10^–6^
P62937	PPIA	Peptidylprolyl Isomerase A	0.7	− 0.53	0.11	8.7 × 10^–6^
Q9Y6B6	SAR1B	Secretion associated Ras related GTPase 1B	0.4	− 4.04	0.89	1.1 × 10^–5^
IPI12345678	–	–	0.5	− 1.09	0.24	1.4 × 10^–5^
P04275	VWF	Von Willebrand factor	2.6	1.09	0.24	1.6 × 10^–5^
Q14956	GPNMB	Glycoprotein Nmb	3.1	3.06	0.69	1.9 × 10^–5^
P32856	STX2	Syntaxin 2	2.1	1.55	0.35	1.9 × 10^–5^

*EVs* extracellular vesicles; *IPF* idiopathic pulmonary fibrosis

^*^Fold change in cases relative to controls in quantitative protein abundance before log transformation

**Fig. 2:**
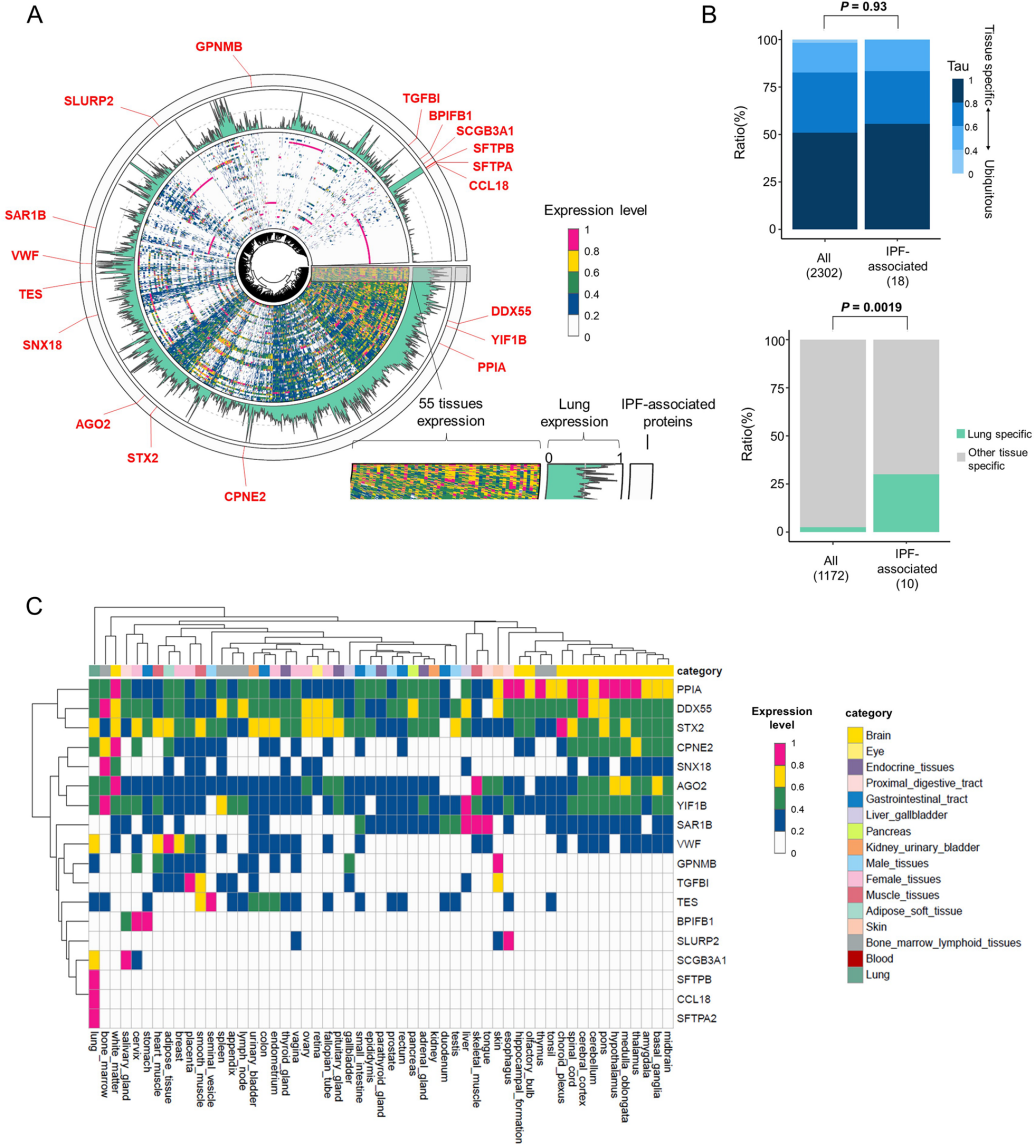
Tissue expression profiles of proteins contained in serum EVs. **A** A circular plot shows the expression levels of 2302 serum EV proteins in 55 tissues (inner edge), expression levels in lungs (middle edge), and IPF-related proteins (outer edge). Tissue expression data were obtained from the Human Protein Atlas. **B** Bar plots show the composition of tissue-specificity metrics (Tau score) (left) and the ratio of lung-specific proteins (right). *P* values were calculated using Fisher's exact test. **C** Heatmap shows tissue expression profiles of the IPF-associated proteins in a similar manner to the inner edge of (**A**). The lung is located in the leftmost of the 55 tissues. EVs, extracellular vesicles; IPF, idiopathic pulmonary fibrosis

**Fig. 3:**
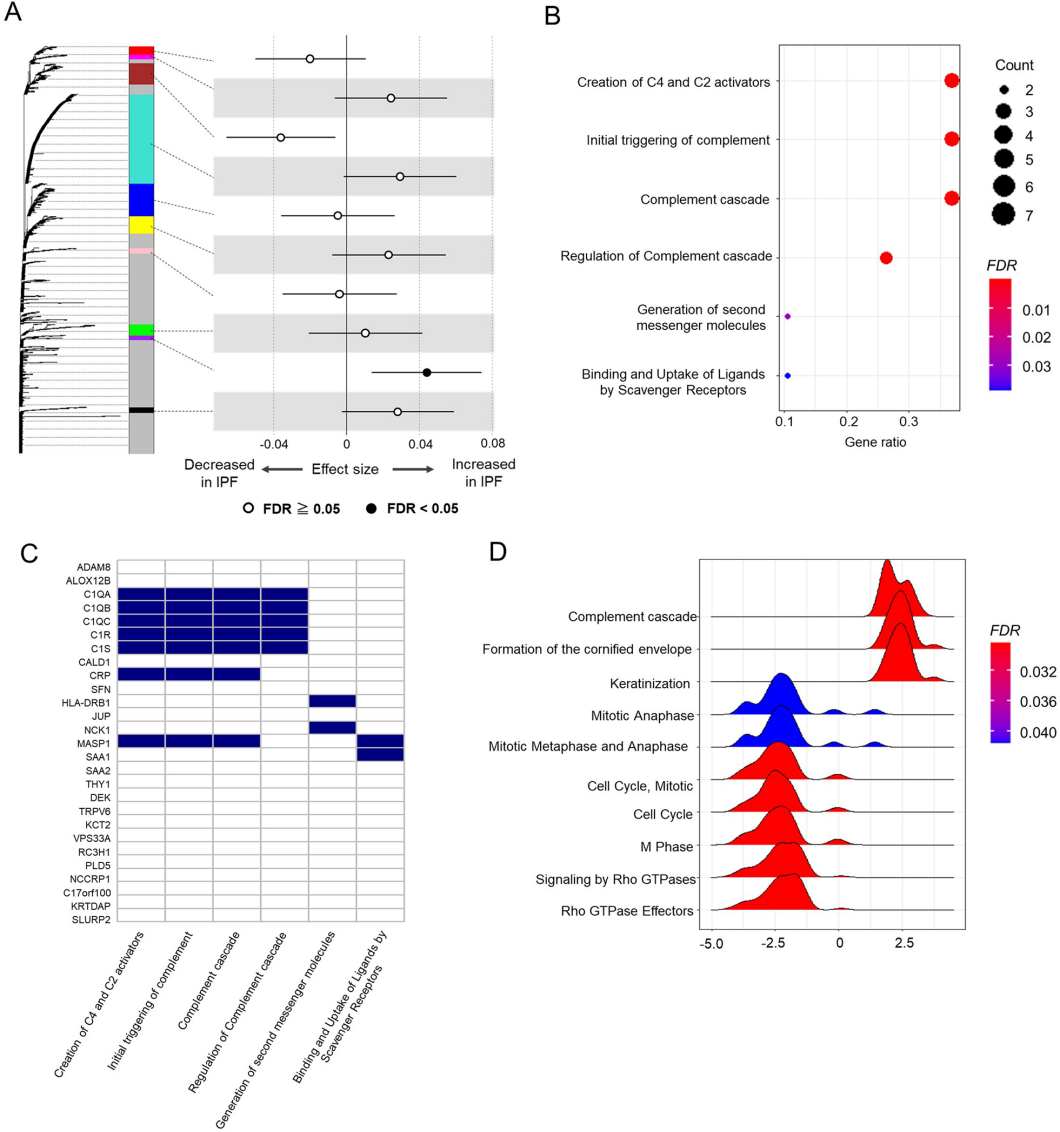
Involvement in complement proteins with IPF. **A** A dendrogram based on protein abundance in serum EVs and 10 protein modules clustered by weighted co-expression network analysis (WGCNA). A forest shows the result of the association test for the protein modules. The dots indicate the effect size of each eigenprotein, and the whiskers represent 95% confidence intervals. **B** The significantly enriched pathways of the purple module by the hypergeometric test for Reactome database. The dot colour indicates the statistical significance of the enrichment, and the dot size represents the gene ratio annotated to each term. **C** A heatmap shows the purple module proteins included in the enriched pathway terms. **D** The top 10 enriched pathways by the gene set enrichment analysis (GSEA) for Reactome database. A ridgeline plot visualizes t statistics distributions in linear regression of core enriched genes for each term. The colour indicates the statistical significance of the enrichment. EVs, extracellular vesicles; IPF, idiopathic pulmonary fibrosis

To remove potential confounding variables, we compared the SFTPB levels between IPF cases and controls after stratifying sex, smoking history, or antifibrotic therapy. This evaluation reaffirmed that SFTPB was increased in IPF even when accounting for these factors (Fig. E4). We also evaluated the superiority of EVs-contained SFTPB over serum SFTPB by analyzing the same serum specimens from which we extracted EVs in the replication cohort. We didn’t observe a significant correlation between the SFTPB levels in serum and EVs (r = 0.26, *P* = 0.092; Fig. 1E), while the serum SFTPB was also increased. Notably, the comparison between cases and controls indicated the potential of EVs-contained SFTPB as a more sensitive biomarker than serum one (*P* = 1.8 × 10^–4^ in EVs and 0.029 in serum, respectively).

**Fig. 4:**
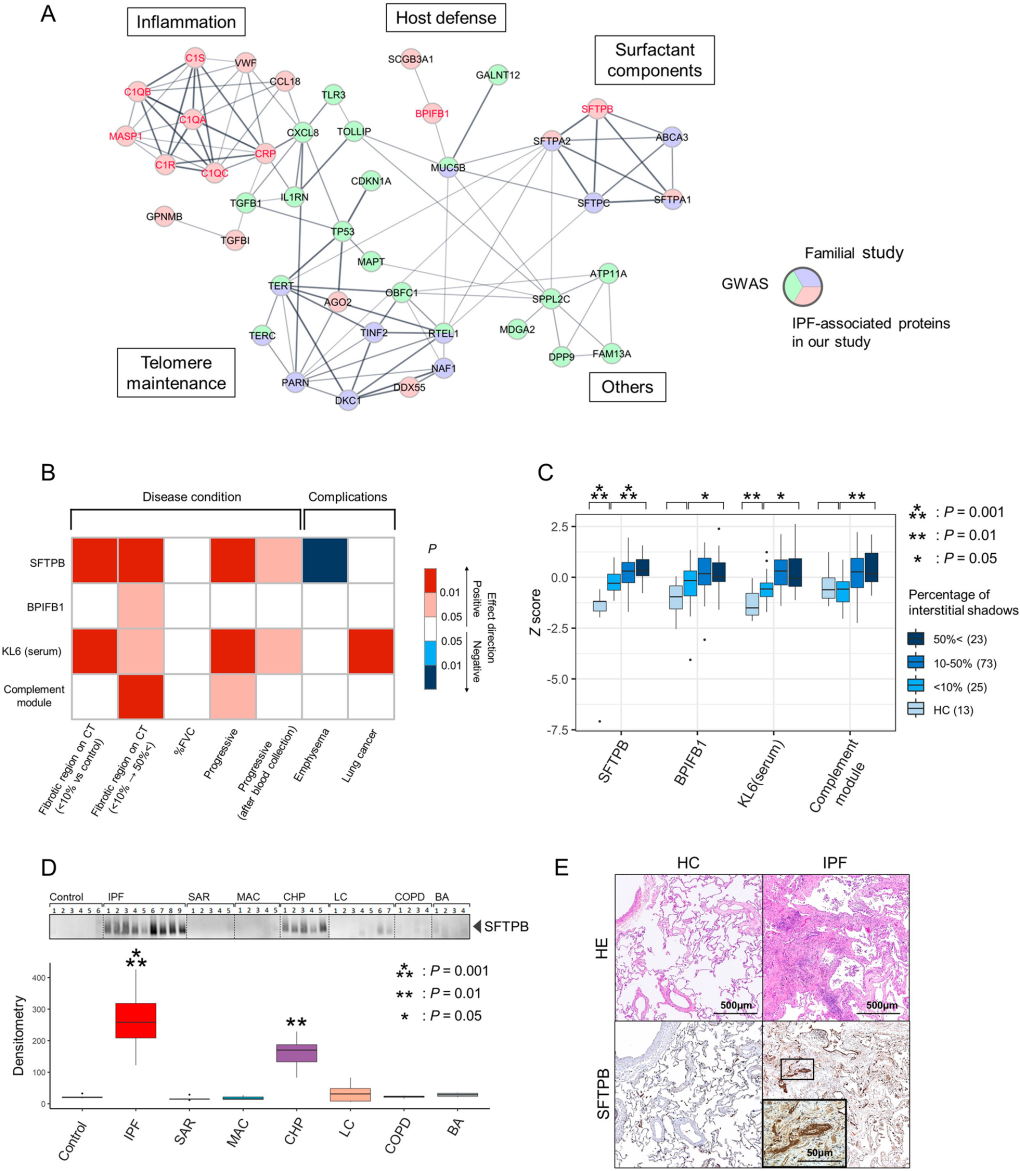
IPF-associated proteins reflect various etiologies and clinical features in IPF. **A** Protein–protein interaction network of IPF-associated proteins in our study and known IPF-associated genes. SFTPB, BPIFB1, and complement modules are indicated by red characters. Circle backgrounds indicate which category the queried gene belongs to among our study, GWAS, and familial studies. The graph edges depict the interaction score in the STRING database. **B** A heatmap indicates the statistical significance of the association test of the IPF-associated proteins and serum KL6 with clinical features. **C** Box plots show the distribution of normalized IPF-associated proteins and serum KL6 levels by the extent of fibrosis on CT imaging. The extent of fibrosis is binned into < 10%, 10–50%, and ˃ 50%, and the number of patients in each category is shown in parentheses. *P* values indicate the significance of the effect size of the CT region on the amount of protein in the linear regression analysis between IPF patients with < 10% fibrosis and healthy controls, or for the expansion of fibrosis within IPF patients. **D** Upper panel: western blot analysis for SP-B in the serum EVs of healthy controls (*n* = 6), IPF (*n* = 9), sarcoidosis (*n* = 5), mycobacterium avium complex (*n* = 5), chronic hypersensitivity pneumonitis (*n* = 5), lung cancer (*n* = 7), chronic obstructive pulmonary disease (*n* = 4), and bronchial asthma patients (*n* = 4). Lower panel: box plots indicating the densitometry distribution of the western blot analysis. *P* values were calculated between healthy controls and individual disease using the Wilcoxon rank-sum test. **E** Histopathology and immunohistochemistry of the lung obtained from an IPF patient and a control participant. The inset magnifies regenerative airway epithelial cells in the tissue with fibrotic remodeling. BPIFB1, BPI Fold Containing Family B Member 1; EVs, extracellular vesicles; IPF, idiopathic pulmonary fibrosis; SFTPB, surfactant protein B

### Tissue expression analysis for serum EV proteins

We evaluated the tissue expression profiles of all proteins in serum EVs because serum EVs are derived from various organs. We referred to the tissue expression data from the Human Protein Atlas for the 2420 proteins in serum EVs and obtained expression data for 2302 proteins (Fig. 2A). Approximately half of the serum EV proteins were tissue-specific (Tau score > 0.8), and their Tau score distribution was comparable with that of the 19 IPF-associated proteins in the discovery cohort (*P* = 0.93; Fig. 2B). Notably, the 19 IPF-associated proteins included more lung-specific proteins than all proteins in serum EVs (*P* = 0.0019). Hierarchical clustering based on tissue expression data described that the IPF-associated proteins tended to be lung-specific (Fig. 2C). The lung-specific proteins included SFTPA2, SFTPB, CCL18, and SCGB3A1. Thus, we demonstrated the tissue expression landscape of serum EV proteins and lung specificity of IPF-associated proteins, successfully capturing lung information via serum EVs.

### Associations between complement proteins and IPF

Protein module analyses were performed to evaluate the aggregated effect of proteins with similar abundance profiles. We identified 10 protein co-expression modules across IPF and healthy controls. One purple protein module was significantly associated with IPF (FDR = 0.048; Fig. 3A). The IPF-associated module contained several proteins that are upstream components of the complement system, including C1QA, C1QB, C1QC, and MASP1. The IPF-associated module was significantly enriched in the complement system in the hypergeometric test of the Reactome pathway (FDR = 3.6 × 10^–15^; Fig. 3B, C). The association between the complement system and IPF was also confirmed in the gene set enrichment analysis (GSEA), which utilized the statistics of all proteins in the serum EVs　(FDR = 0.028; Figs. 3D, E5).

**Fig. 5:**
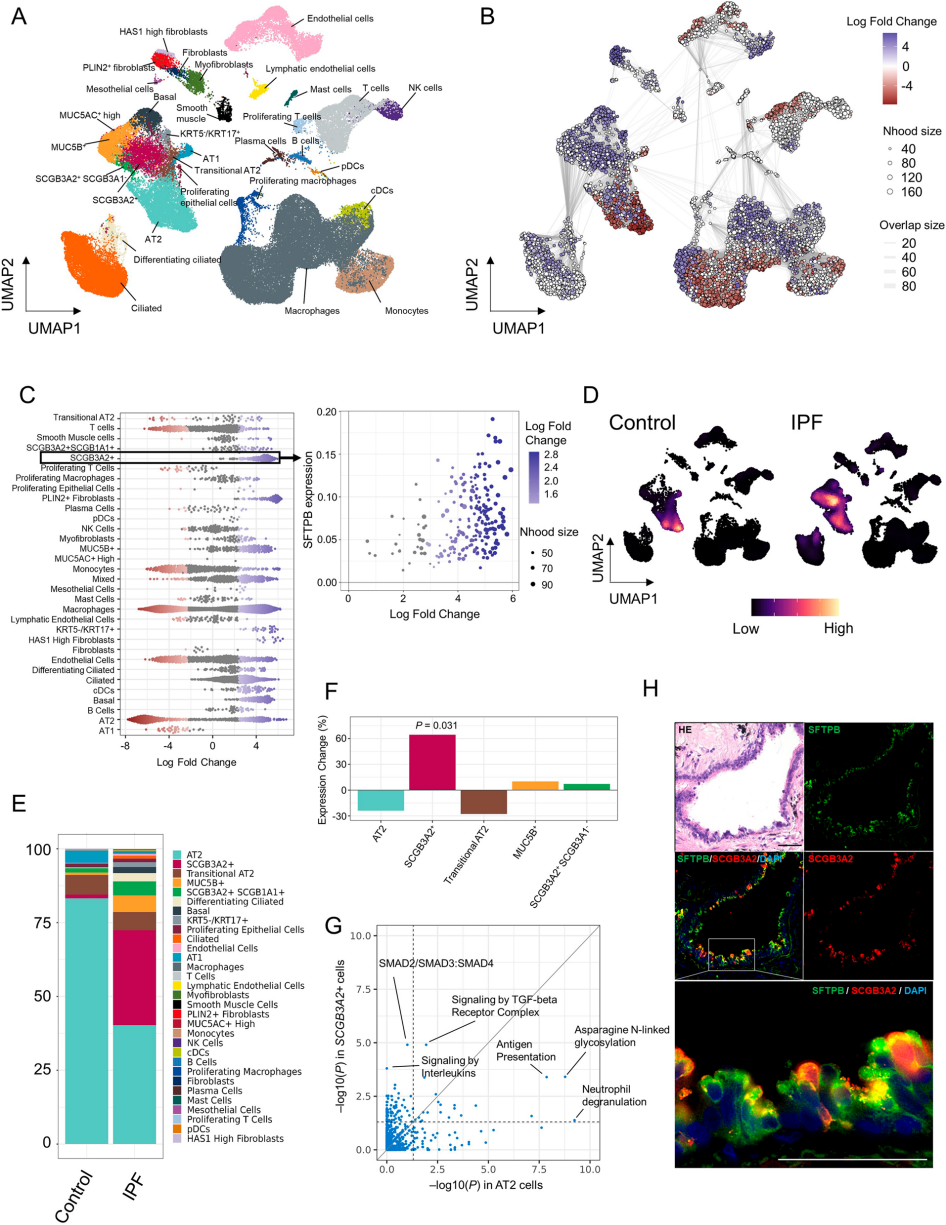
Increased *SFTPB* is associated with TGF-β/SMAD pathway in *SCGB3A2* + cells. **A** Uniform Manifold Approximation and Projection embedding of jointly analyzed single-cell transcriptomes from 89,326 cells from 12 IPF and 10 control lungs. **B** A neighborhood graph of the results from Milo differential abundance testing. Nodes are neighborhoods coloured by their log_2_ fold change. Non-differential abundance neighborhoods (FDR ≥ 0.1) are coloured with white. The graph edges depict the number of cells shared between the neighborhoods. **C** A beeswam plot shows the distribution of log_2_ fold change in abundance in the neighborhood between IPF and control lungs for each of the 32 cell types (left). Colours are represented as (**B**). A scatter plot for *SCGB3A2* + cells was extracted from the total beeswam plot, and the average *SFTPB* expression of the cells in each neighborhood is mapped on the y-axis (right). **D** Projection of gene expression density of *SFTPB* in IPF and control lungs. **E** Bar plots show the composition of the cellular origin of *SFTPB* gene expression. **F** Expression changes in the differential expression analysis are shown for the five major *SFTPB* source cell types. The *P* values with a significant association are indicated. **G** A scatter plot shows the statistical significance of the enrichment analysis for AT2 cells (x-axis) and *SCGB3A2* + cells (y-axis) in IPF lungs, respectively. The vertical and horizontal dashed Line indicates the threshold of 0.05. **H** Immunofluorescence staining of an IPF lung for SFTPB (green), SCGB3A2 (red), and DAPI (blue). Scale bar = 50 μm. IPF, idiopathic pulmonary fibrosis; SFTPB, surfactant protein B

### Pathways and clinical features involving IPF-related proteins

We investigated the functional associations among the 19 IPF-associated proteins, complement proteins, and known IPF-associated genes by querying the STRING database (Fig. 4A). Known IPF-associated genes were classified into five categories based on their biological pathways: surfactant components, host defense in secretions, inflammation, telomere maintenance, and others. Our identified proteins and protein modules were associated with genes in various categories, implying that they reflect various aspects of IPF etiologies. We observed that IPF-proteins belonged to various biological categories: SFTPB in surfactant components, BPIFB1 and MUC5B in host defense in secretions, and complement system proteins and CXCL8 in inflammation. The association between our identified proteins and various IPF etiologies motivated us to explore the heterogeneity among IPF patients. We compared the levels of SFTPB, BPIFB1, the complement module, and serum KL6 in IPF cases and observed heterogeneity in abundance profiles (Fig. E6A). This result was also reflected in the moderate correlations between these proteins (Fig. E6B); the maximum correlation estimate was 0.5 (between SFTPB and BPIFB1), and the minimum was 0.11 (between SFTPB and complement module). These results indicate that our identified proteins could capture various aspects of heterogeneous IPF patients.

We evaluated the association of these proteins with the following clinical features: extent of fibrosis on CT images, %FVC, %DLCO, progressive phenotype, and complications of lung emphysema or lung cancer (Fig. 4B). SFTPB was positively associated with the fibrotic region on CT (*P* = 2.4 × 10^–4^ in the early stage against control and *P* = 3.7 × 10^–4^ in the expansion of fibrotic region) and progressive phenotype (*P* = 0.0074) and negatively associated with %FVC (*P* = 0.034) and emphysema complications (*P* = 0.0081). Complications of emphysema led to decreased SFTPB levels, but the presence of fibrosis remained a marked increased factor of SFTPB compared to controls (Fig. E7). While serum KL6 levels were positively associated with lung cancer complication (*P* = 0.0095), SFTPB in EVs was not (*P* = 0.37). BPIFB1 was positively associated with only the CT region (*P* = 0.017), and the complement module was positively associated with the CT region (*P* = 0.0015) and progressive phenotype (*P* = 0.043). When we focused our analysis between the IPF patients who met the progression criteria within two years after blood collection and non-progressive IPF, SFTPB and serum KL6 remained significant while the power was decreased due to the reduction of sample size (*P* = 0.018 and 0.036, respectively). The expressions of all the proteins tended to increase in the early stage of IPF and increased linearly in response to the expansion of the fibrotic region, with SFTPB being the most strongly associated (Fig. 4C). Furthermore, all these proteins were negatively associated with %DLCO (*P* < 0.01). Previous reported IPF-associated proteins also tended to be associated with the clinical profiles of IPF, but the significance of SFTPB was superior to those of them (Table E3).

To confirm the robustness of the SFTPB results and compare them with other respiratory diseases, we performed immunoblotting of serum EVs. Consistent with the proteome findings, SFTPB expression was increased in conditions characterized by fibrosis, such as IPF and chronic hypersensitivity pneumonitis (CHP) (Fig. 4D), with IPF showing a higher expression of SFTPB compared with CHP. SFTPB expression in non-fibrotic diseases, such as lung cancer or chronic obstructive pulmonary disease, was not increased, indicating that SFTPB could be a specific biomarker for fibrosis. In the immunohistochemistry of the IPF lung, expression of SFTPB was upregulated in the airway epithelial cells, alveolar epithelial cells, and macrophages (Fig. 4E). Notably, we observed strong positive signals in regenerative airway epithelial cells in the tissue with fibrotic remodeling (magnified in the inset of Fig. 4E). Thus, we confirmed that serum EVs could capture multiple proteins involved in IPF genetic components and that they were associated with multiple clinical features of IPF; among these proteins, SFTPB was most significantly related to the disease activity.

### Primary cells producing SFTPB in IPF lungs

We evaluated the biological perspective of SFTPB by analyzing the publicly available single-cell dataset containing 89,326 cells from 12 IPF and 10 control lungs (Fig. 5A). To reveal the compositional changes between IPF and controls, we applied Milo to identify 7,115 neighborhoods, among which 4,329 showed evidence of differential abundance (FDR < 0.1, Fig. 5B). *SCGB3A2* + cell cluster contained significantly increased numbers of neighborhoods in IPF lungs (Fig. 5C). Furthermore, the increased *SCGB3A2* + cells expressed higher levels of *SFTPB*. While type 2 alveolar epithelial (AT2) cells expressed more *SFTPB* in the control lungs, the *SCGB3A2* + cells were the primary source of *SFTPB*, in addition to AT2 cells, in the IPF lungs (Fig. 5D). The pseudobulk assessment by summing expression levels per cell clearly depicted the changes in cell types expressing *SFTPB* between the IPF and control lungs (Fig. 5E). Differential expression analysis also demonstrated increased *SFTPB* expression in the IPF lungs with *SCGB3A2* + cells (fold change = 1.64, *P* = 0.031; Fig. 5F). We used hdWGCNA to identify the *SFTPB* co-expression genes in the *SCGB3A2* + and AT2 cells in IPF lungs. Notably, *SFTPB* co-expression in the *SCGB3A2* + cells included many genes involved in the TGF-β/SMAD pathway, such as *SMAD3*, *MYC*, *WWTR1*, and *E2F4*. Correspondingly, the *SFTPB* co-expression module in the *SCGB3A2* + cells was most significantly enriched in the TGF-β/SMAD pathway but not in the AT2 cells (Fig. 5G): “Transcriptional activity of SMAD2/SMAD3:SMAD4 heterodimer” (FDR = 1.2 × 10^–5^ in *SCGB3A2* + cells and FDR = 0.098 in AT2 cells) and “Signalling by TGF-β Receptor complex” (FDR = 1.2 × 10^–5^ in *SCGB3A2* + cells and FDR = 0.012 in AT2 cells), respectively. To demonstrate the robustness of the scRNA-seq analysis, *SFTPB* + *SCGB3A2* + cells were confirmed using the IPF lung tissue sample (Fig. 5H). Thus, the single-cell analysis revealed that *SFTPB* was associated with the TGF-β/SMAD pathway, specifically in the *SCGB3A2* + cell cluster, which increased the cell population in IPF lungs.

We extended our analysis to 55 IPF and 52 healthy lung scRNA-seq dataset from the Human Lung Cell Atlas [34] to validate the transition in the cellular sources of *SFTPB* (Fig. E9A). This broader dataset supported our findings that *SFTPB* originating from secretory airway cells including *SCGB3A2* + cells was increased in IPF lungs, while *SFTPB* from AT2 cells was decreased (Fig. E9B, C).

Finally, we investigated the association between SFTPB and SCGB3A2 using our proteomics data (Fig. E10). While there was no association between SFTPB and SCGB3A2 in healthy subjects (effect size = − 0.11, *P* = 0.55), SFTPB was positively associated with SCGB3A2 in IPF (effect size = 0.52, *P* = 9.5 × 10^–4^). This finding supports the observation from the scRNA-seq analysis that SFTFP in IPF is associated with *SCGB3A2* + cells.

## Discussion

In this study, our comprehensive proteomic analysis of serum EVs revealed that several lung-specific proteins and upstream components of the complement system were significantly associated with IPF. We used DIA, a non-targeted proteomic method, to comprehensively and repeatedly sample every peptide in the protein digest, producing a wide identification breadth and reproducible quantification compared with conventional LC–MS/MS analysis [40]. By adopting this technology for EVs, which are less susceptible to serum contaminants, we took full advantage of the tissue-derived messages from the serum.

SFTPB, the most associated protein in our study, is a lipid-protein complex essential for stabilizing the delicate structure of mammalian alveoli by reducing the surface tension at the air–liquid interface [41]. Pulmonary surfactant proteins are classified into four families: SFTPA, SFTPB, SFTPC, and SFTPD. Although the three surfactant proteins other than SFTPB are IPF-associated genes and candidate molecular biomarkers for IPF [39], the association between SFTPB and IPF remains limited. Recently, serum SFTPB levels were reported to be increased in patients with interstitial lung abnormalities (ILA) and associated with ILA progression through aptamer-based proteomics [42], although the increased SFTPB expression was not confirmed by immunoblotting. While aptamer-based proteomics is a targeted approach, LC–MS/MS is a non-targeted approach. Their roles are complementary, and confirmation of both results makes proteomic analysis robust [43]. Another study reported that C-proSP-B in serum was increased in IPF patients [44]. Our findings were not only consistent with those of these previous studies but also added novel findings for SFTPB in terms of clinical and biological perspectives. Previous studies have reported that SFTPB levels are elevated in interstitial lung abnormalities (ILAs) or in patients with IPF compared to healthy individuals [42, 44]; however, they did not evaluate its relationship with disease activity within the IPF population. In contrast, our study highlights that EV-contained SFTPB correlates with disease progression and key clinical indicators, suggesting its utility not only as a diagnostic marker but also as a tool for monitoring disease severity and activity in IPF. The progressive fibrosing phenotype of interstitial lung diseases (ILDs), such as progressive pulmonary fibrosis (PPF) or progressive fibrosing interstitial lung disease (PF-ILD), has received increasing attention [23, 45]. Considering that SFTPB in EVs could be a better biomarker for diagnosis or disease severity in IPF and IPF is a representative disease entity among PF-ILD, further investigation should assess the potential of SFTPB for PPF or PF-ILD.

This study revealed that the *SCGB3A2* + cell population was a primary source of *SFTPB* in IPF lungs. *SCGB3A2* is a marker heterogeneously expressed in secretory epithelial cells, including club cells [46], and the *SCGB3A2* + club cell subpopulation was altered in IPF lungs, with increased expression of mucins, cytokine, and extracellular matrix genes [47]. Although increased expression of SFTPB outside the lungs simply represents the destruction of the alveolar-capillary membrane in other conditions [48, 49], increased SFTPB levels in serum EVs in IPF may reflect the convergence of alveolar and secretory programs which have been described in the murine lung [29, 50]. Such changes in cell lineage contributions to SFTPB production can provide valuable insights into the cellular dynamics and pathophysiology of lung fibrosis.

BPIFB1, another promising biomarker, is secreted by the goblet cells and minor glands of the respiratory and upper aerodigestive tracts [51]. BPIFB1 was reported to be upregulated in ILA [42] and IPF [52, 53]. Our analysis not only confirmed a robust association between BPIFB1 and IPF but also revealed that BPIFB1 amounts in EVs were increased in response to worsening CT imaging. Given its role as a trigger in the innate immune system against bacteria in the airways as well as the representative risk gene MUC5B [54], BPIFB1 can potentially reflect the activity of an innate immunity pathway in IPF etiologies.

Regarding innate immunity, a protein module including upstream components of the complement system was associated with CT region and a progressive phenotype. The complement protein C1q activates lung fibroblasts/epithelial cells and induces pulmonary fibrosis in mice [55], and high complement protein C1q levels in pulmonary fibrosis are associated with poor prognosis [56]. C1q reportedly activates Wnt signaling [57] for the differentiation of mesenchymal stem cells in fibrotic lung disease [58]. In addition to being a potential diagnostic and prognostic biomarker, complement proteins can be therapeutic targets because the complement system can be modified by interventions, such as inhibition by neutralizing antibodies.

Notably, the expressions of SFTPB, BPIFB1, and the complement system were increased in IPF patients from the early disease stages, suggesting their importance for early diagnosis. These proteins are related to IPF causative genes in various etiologies and show heterogeneous profiles within the IPF group. This observation may reflect the differences in etiologies among the patients with IPF and provide the possibility of stratifying IPF patients based on multiple biomarkers that reflect distinct pathogenic mechanisms, moving beyond traditional case–control comparisons performed in previous studies.

We also described the landscape of the tissue expression profile of serum EVs. Our tissue expression analysis revealed that approximately half of the proteins in serum EVs were tissue-specific proteins, and IPF-associated proteins were enriched in lung-specific proteins. This indicates that our strategy of obtaining lung information via serum EVs is effective.

Despite the great advantage of DIA of EVs for efficient discovery of biomarkers, this study has some limitations. First, the application of EVs as a liquid biopsy in a clinical setting is fascinating; however, there is a problem with the simplicity of EV extraction for its direct analysis at the bedside. Future efforts should aim to simplify and automate EV isolation methods to facilitate routine clinical use. Second, we did not directly validate the proteins in serum EVs were produced from specific cells in lungs. Identifying the cellular origins of proteins in serum EVs is challenging with even current technology. The use of advanced technologies such as spatial transcriptomics or EV surface proteomics may enable more precise tracing of EV contents to their cellular sources. Finally, given that genome, transcriptome, and metabolome networks are also important for precision medicine, it would be intriguing to aggregate our data by integrating further multiple omics. In particular, our findings suggest that SFTPB has the potential to be used as a biomarker for predicting disease progression in IPF, and prospective studies are needed to validate its prognostic utility. Moreover, the heterogeneous elevation of lung- and complement-related proteins observed in serum EVs may provide a basis for stratifying IPF patients into molecular subtypes, paving the way for more personalized therapeutic approaches. Such a system biology platform using clinical data, omics, and bioinformatics may help unravel the complexity of IPF and other ILD in the future.

## Conclusions

Our study demonstrated that serum EVs can capture a heterogeneous set of proteins—including lung-specific and complement-related components—that reflect the underlying diversity of disease mechanisms in IPF and may enable patient stratification. Among these proteins, SFTPB showed strong associations with multiple clinical features indicative of fibrotic progression. We also showed that SFTPB was derived from SCGB3A2⁺ secretory epithelial cells enriched in TGF-β/SMAD signaling, suggesting its biological relevance to fibrotic remodeling in IPF. Our strategy provides the possibility of diagnostic and therapeutic stratification within heterogeneous IPF for implementing personalized medicine.

## Supplementary Information

Supplementary Material 1

## Data Availability

The proteome data are available upon reasonable request. The raw and processed 10X Genomics data we used in scRNA-seq analysis can be downloaded on GEO using accession number GSE135893. The processed cellranger data in Human Lung Cell Atlas can be downloaded via cellxgene (https://cellxgene.cziscience.com/collections/6f6d381a-7701-4781-935c-db10d30de293).
