# Supplementary material for: Serum vesicle biomarkers reflect the disease activity of idiopathic pulmonary fibrosis
Source: J Transl Med. 2025 Oct 15;23:1102. doi: 10.1186/s12967-025-07011-2 (PMC12522894; doi:10.1186/s12967-025-07011-2)
Supplement: Supplementary file 1 — Supplementary Material 1 [file 12967_2025_7011_MOESM1_ESM.docx]

**Serum vesicle biomarkers reflect the clinical features of idiopathic pulmonary fibrosis.**

Online Data Supplement

Yuya Shirai, Takatoshi Enomoto, Yoshito Takeda, Ryuya Edahiro, Miho Takahashi-Itoh, Yoshimi Noda, Yuichi Adachi, Mana Nakayama, Takahiro Kawasaki, Taro Koba, Yu Futami, Hanako Yoshimura, Saori Amiya, Reina Hara, Makoto Yamamoto, Daisuke Nakatsubo, Yasuhiko Suga, Maiko Naito, Kentaro Masuhiro, Takanori Matsuki, Haruhiko Hirata, Kota Iwahori, Izumi Nagatomo, Kotaro Miyake, Shohei Koyama, Kiyoharu Fukushima, Takayuki Shiroyama, Yujiro Naito, Shinji Futami, Yayoi Natsume-Kitatani, Naoko Ose, Soichiro Funaki, Satoshi Nojima, Shigeyuki Shichino, Masahiro Yanagawa, Yasushi Shintani, Mari Nogami-Itoh, Jun Adachi, Yoshikazu Inoue, Takeshi Tomonaga, Yukinori Okada, Kenji Mizuguchi, and Atsushi Kumanogoh.

**Table of contents**

**▪Supplementary Figures…………………………………………………………………………3**

1. Quality check of serum extracellular vesicles obtained from IPF and healthy controls.....3
2. Principal-component analysis using 19 IPF-associated proteins in serum EVs................4
3. Pathway enrichment analysis for 19 IPF-associated proteins...........................................5
4. SFTPB amounts in EVs stratified by the basic clinical features........................................6
5. GSEA result of complement cascade in Reactome database...........................................7
6. Heterogeneous profiles of IPF-associated proteins and serum KL6 in IPF……………….8
7. SFTPB amount stratified in EVs by the presence or absence of emphysema …………...9
8. Distributions of aggregated SFTPB normalised counts by each cell type…………....…..11
9. Validation analysis for SFTPB expressing cells in Human Lung Cell Atlas………..…..…12
10. Comparison between SFTPB and SCGB3A2 in serum EVs……………………..…....…13

**▪Supplementary Tables…………………………………………………………………………15**

1. Quality check of serum extracellular vesicles obtained from IPF and healthy controls...15
2. Diagnostic performance of IPF-associated proteins and serum KL6……….……............16
3. Results of association tests for clinical features of IPF……………...……………........17,18

**Figure E1. Quality check of serum extracellular vesicles obtained from IPF and healthy controls**

**
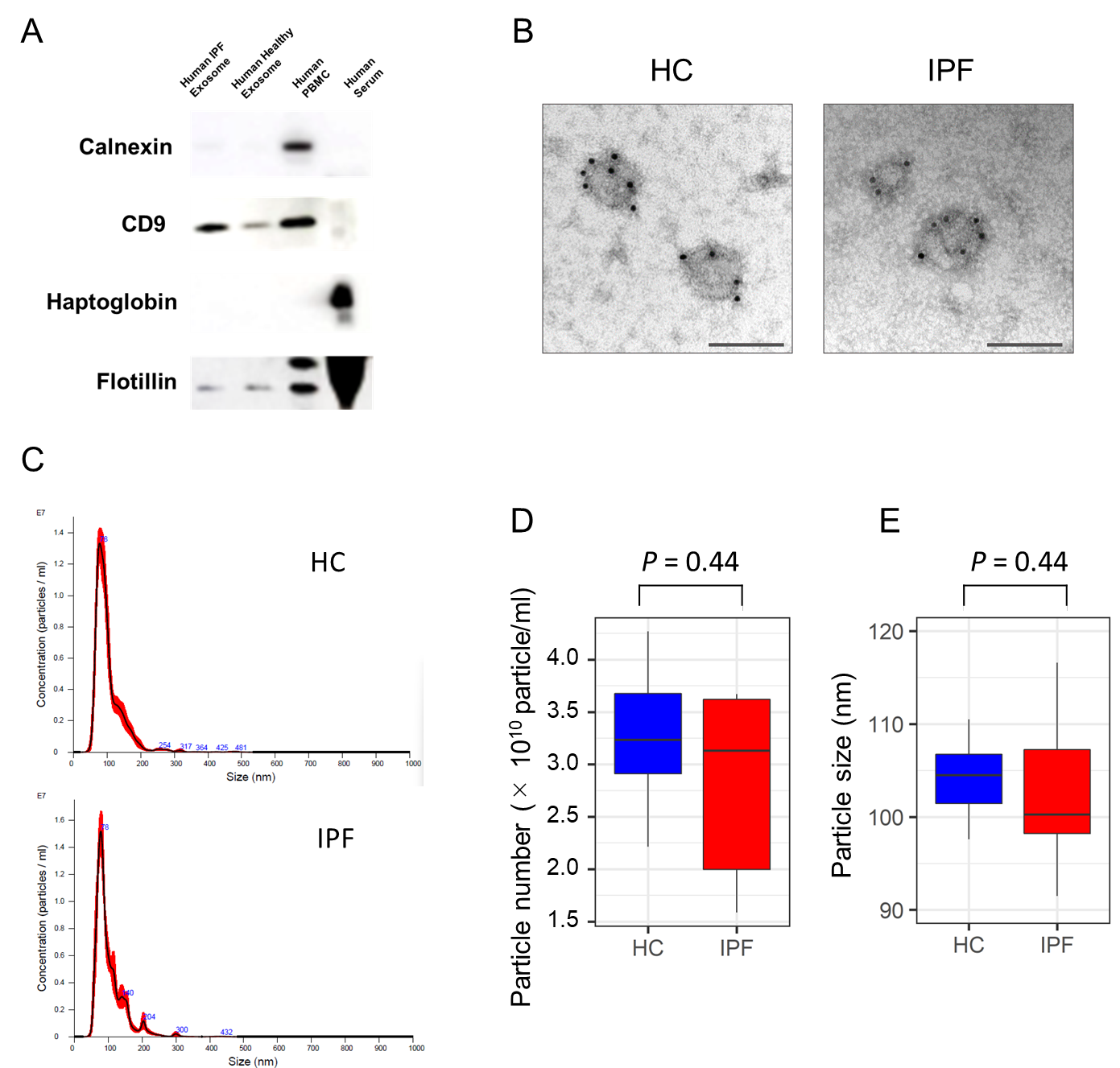
**

(**A**) Immunoblots comparing calnexin, CD9, haptoglobin, and flotillin levels in the serum extracellular vesicles (EVs) versus serum and pbmc. (**B**) The transmission electron microscopic images of serum EVs from a healthy control (HC) and a patient with idiopathic pulmonary fibrosis (IPF); immunogold labelling with CD9. Scale bar=100 nm. (**C**) Representative figures of the particle size distribution curve of serum EVs from an IPF patient and a healthy control using NanoSight. (**D,E**) Box plots describing the numbers and mean diameters of serum EVs analysed using NanoSight from IPF patients and healthy controls. *P*-values were calculated using the Wilcoxon rank-sum test.

**Figure E2. Principal-component analysis using the 19 IPF-associated proteins in serum EVs**

**
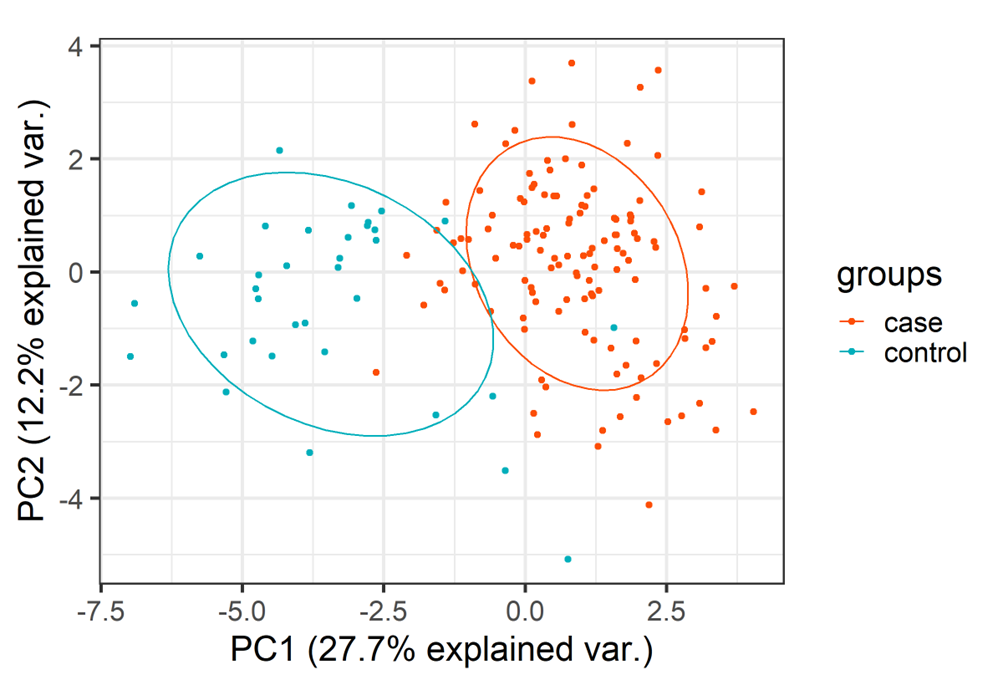
**

A scatter plot of the case-control classes based on the first two principal components of the 19 IPF-associated proteins. The size of the ellipses in normal probability is 0.68. EVs, extracellular vesicles; IPF, idiopathic pulmonary fibrosis

**Figure E3. Pathway enrichment analysis for the 19 IPF-associated proteins**


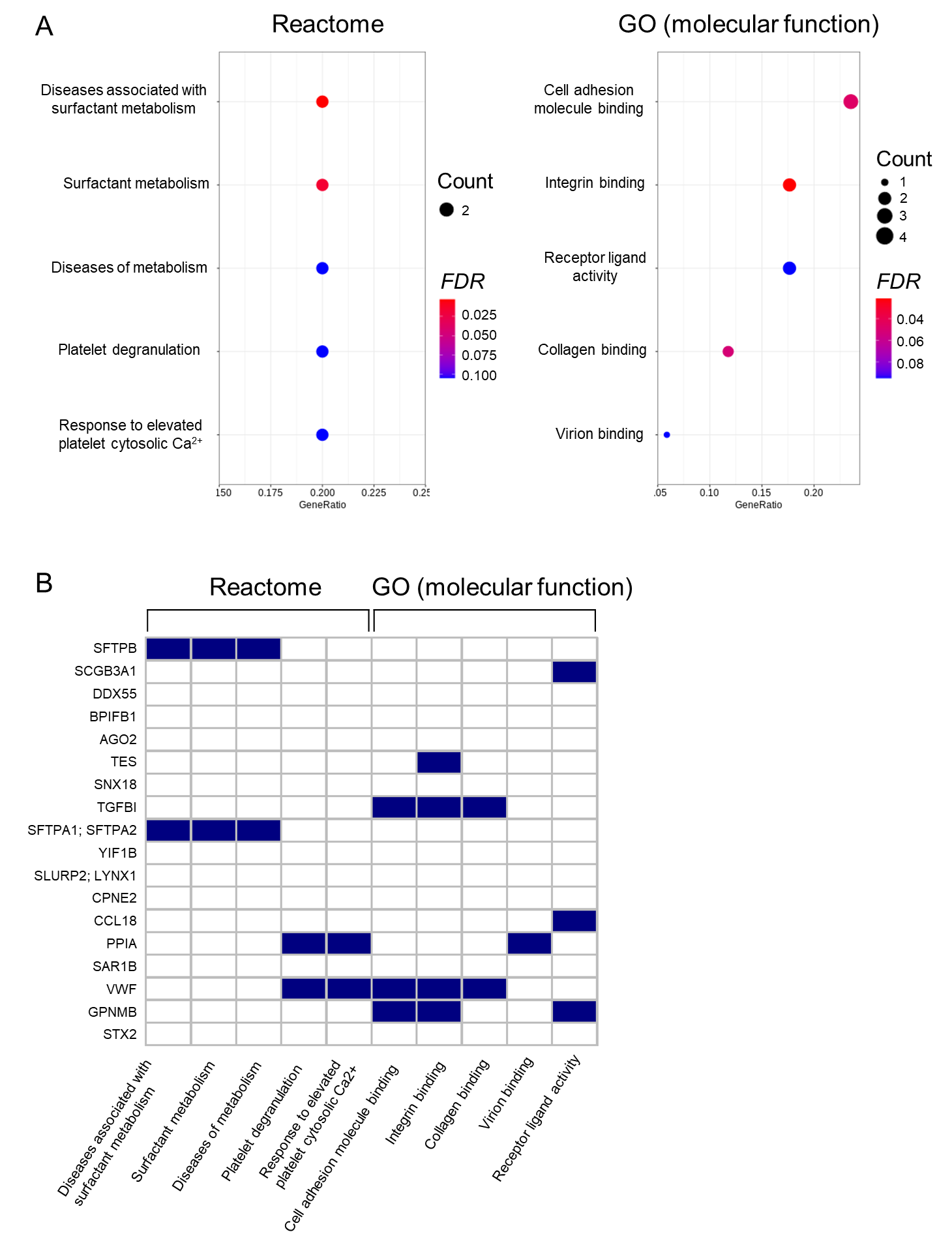


(**A**) The top five enriched pathways according to the hypergeometric test for Reactome database and GO term. The colour of the dot indicates the statistical significance of the enrichment, and its size represents the gene ratio annotated to each term. (**B**) A heatmap shows which IPF-related proteins are included in the enriched pathway terms. FDR, false discovery rate; GO, gene ontology; IPF, idiopathic pulmonary fibrosis

**Figure E4. SFTPB amounts in EVs stratified by the basic clinical features**


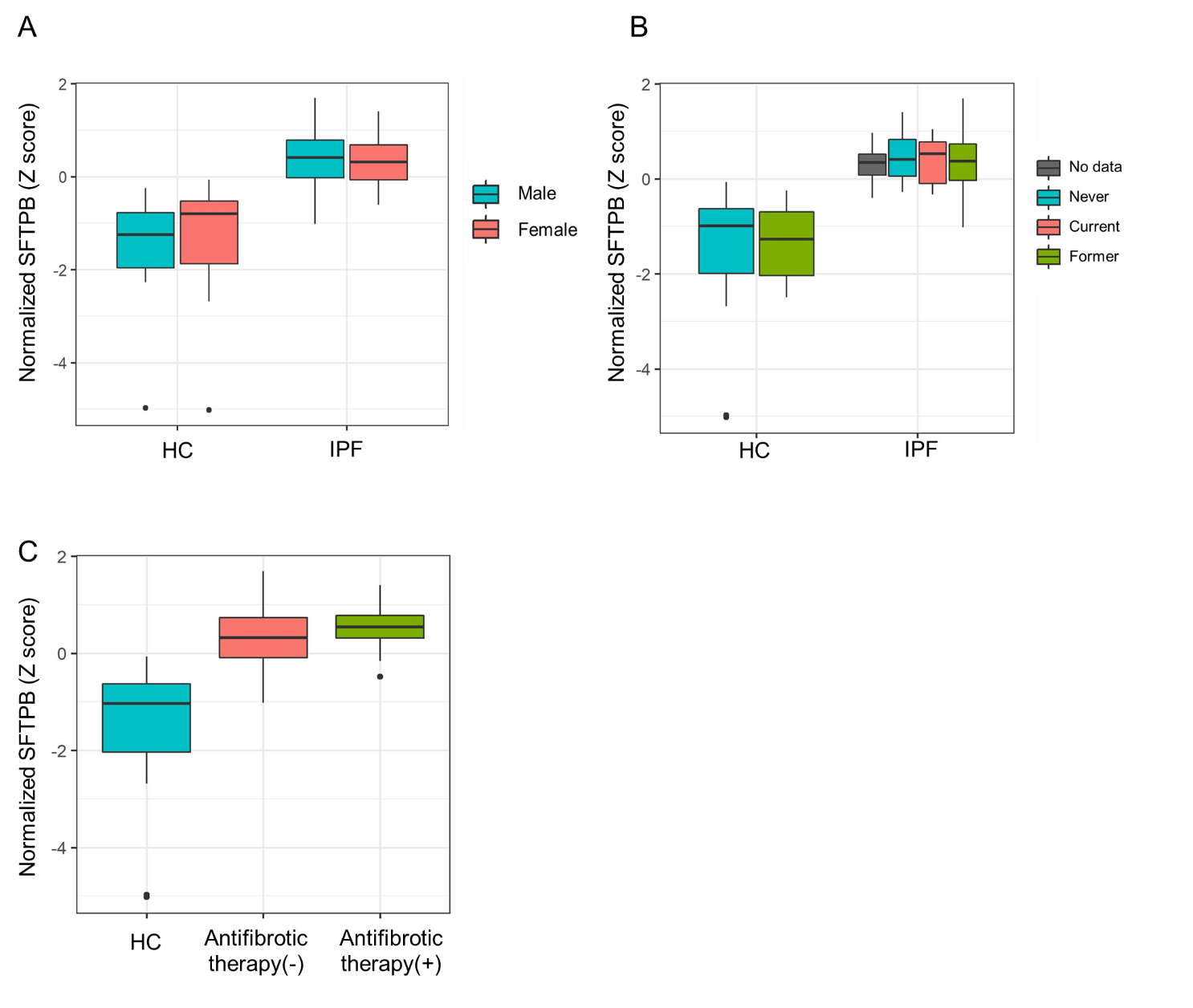


SFTPB is stratified by sex (**A**), smoking history (**B**), and antifibrotic therapy (**C**).

*P* values were calculated by the Wilcoxon rank-sum test.

**Figure E5. GSEA result of the complement cascade in the Reactome database**


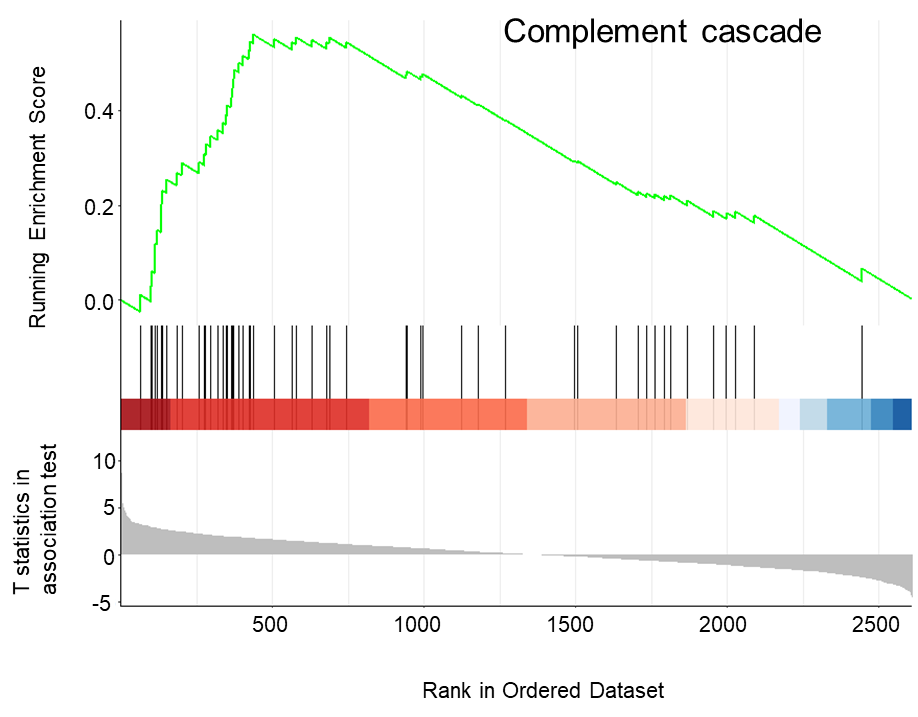


Enrichment plot of the complement cascade in GSEA. The top portion of the plot shows the running enrichment score for the gene set as the analysis walks down the ranked list. The middle portion of the plot shows where the members of the gene set appear in the ranked list of genes. The bottom portion of the plot shows the value of t statistics obtained from the case-control association test of individual protein as input. GSEA, gene set enrichment analysis

**Figure E6. Heterogeneous profiles of the IPF-associated proteins and serum KL6 in IPF**

**
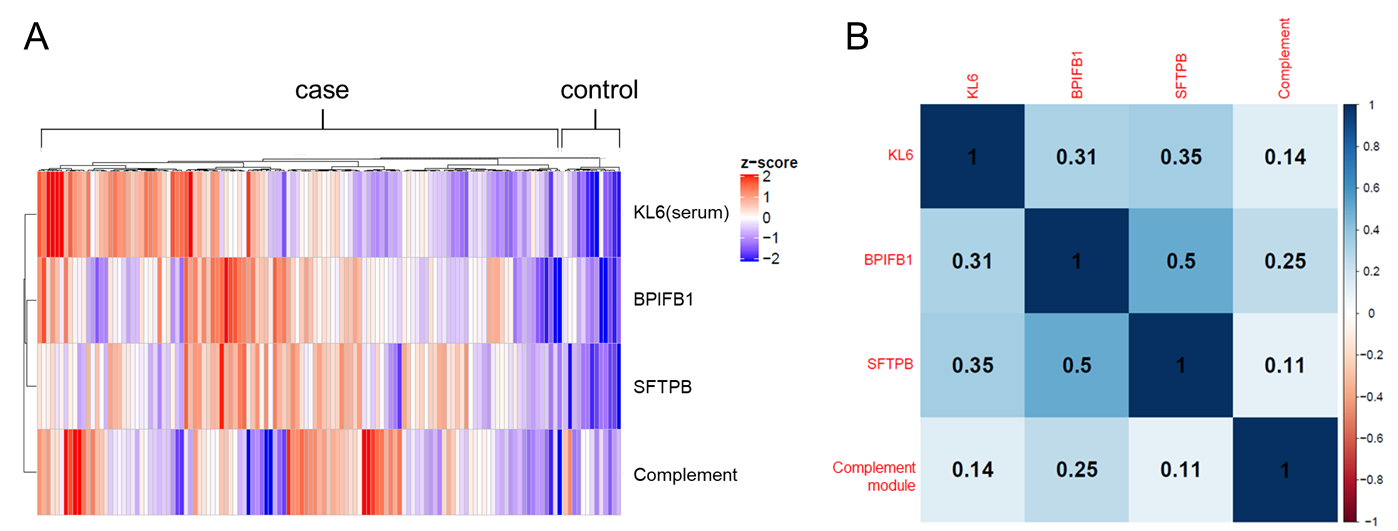
**

(**A**) A heatmap describing SFTPB, BPIFB1, complement module eigenprotein in serum EVs, and serum KL6 levels. Hierarchically clustering is applied to the row against proteins and the column against samples per case and control. (**B**) Correlation matrix between the protein levels of (A). BPIFB1, BPI Fold Containing Family B Member 1; EVs, extracellular vesicles; IPF, idiopathic pulmonary fibrosis; SFTPB, surfactant protein B

**Figure E7. SFTPB amount stratified in EVs by the presence or absence of emphysema**


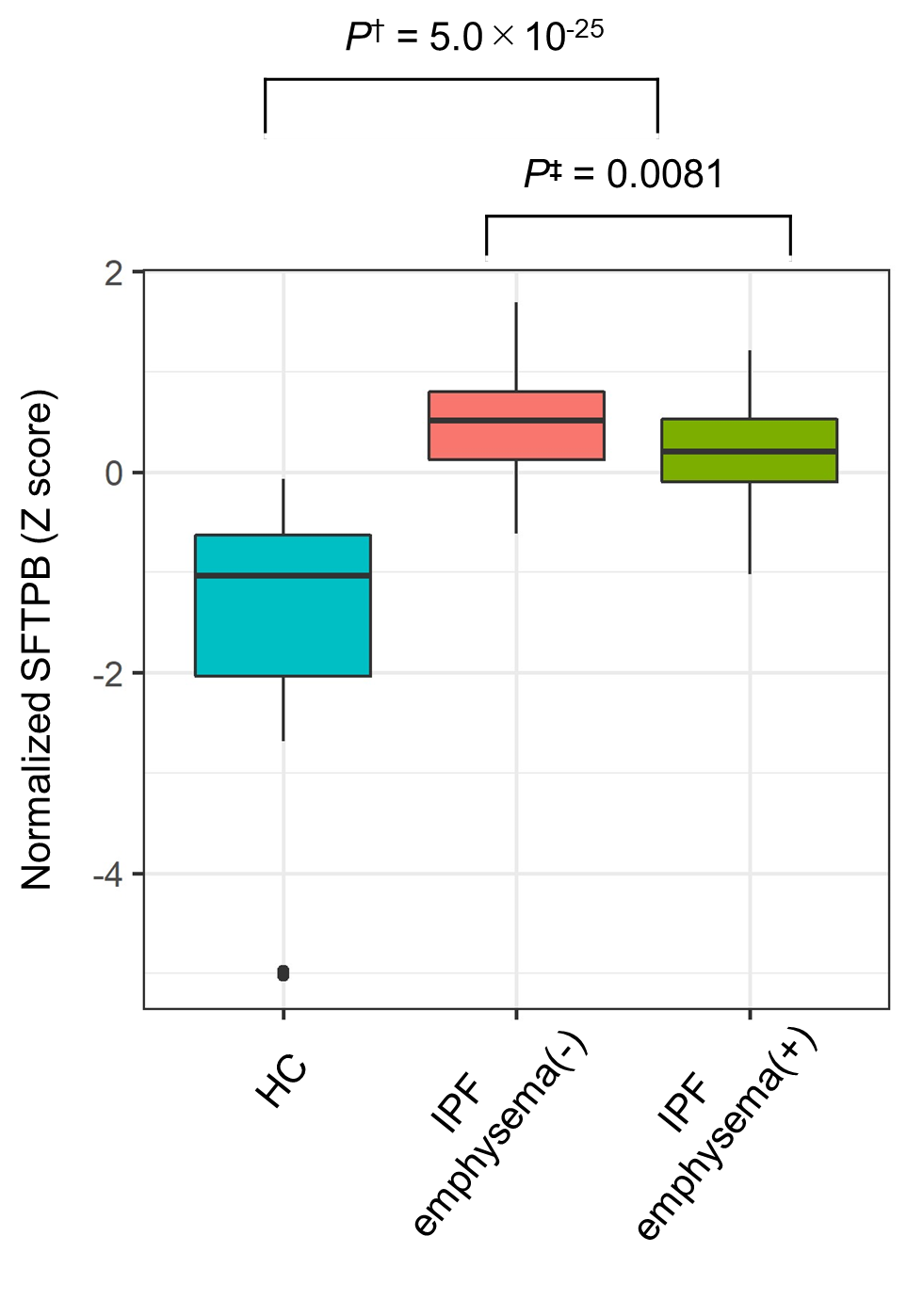


^†^Association analysis between IPF and HC.

**^‡^**Association analysis for emphysema.

**Figure E8. Distributions of the aggregated SFTPB normalised counts by each cell type**


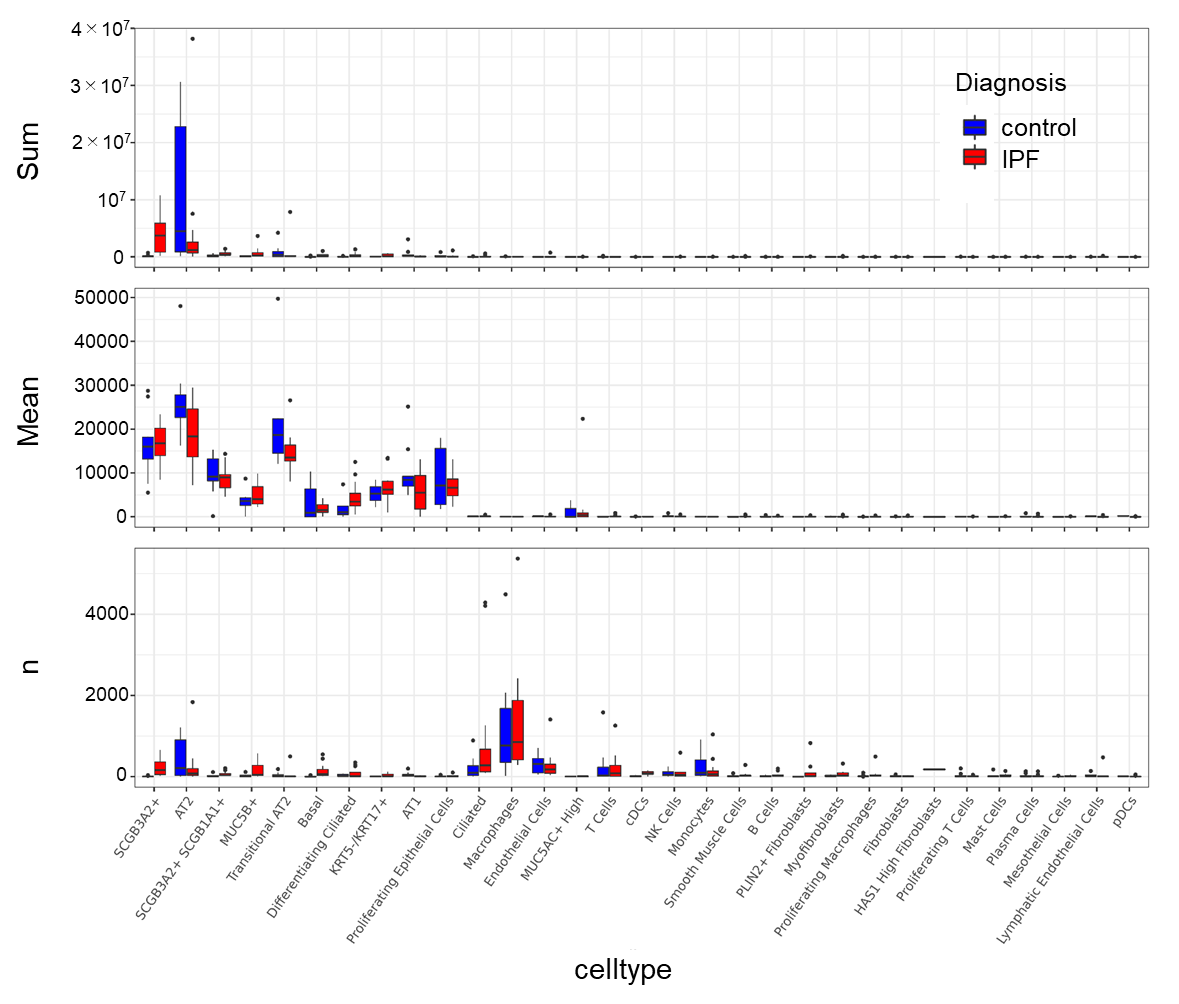


Upper) The distribution of the aggregated count per million (CPM) per each cell type.

Middle) The distribution of the mean CPM per each cell type.

Lower) The distribution of the cell counts per each cell type.

The cell types are arranged in descending order of median of sum in IPF (upper graph). IPF, idiopathic pulmonary fibrosis; SFTPB, surfactant protein B

**Figure E9.** **Validation analysis for SFTPB expressing cells in human lung cell atlas**


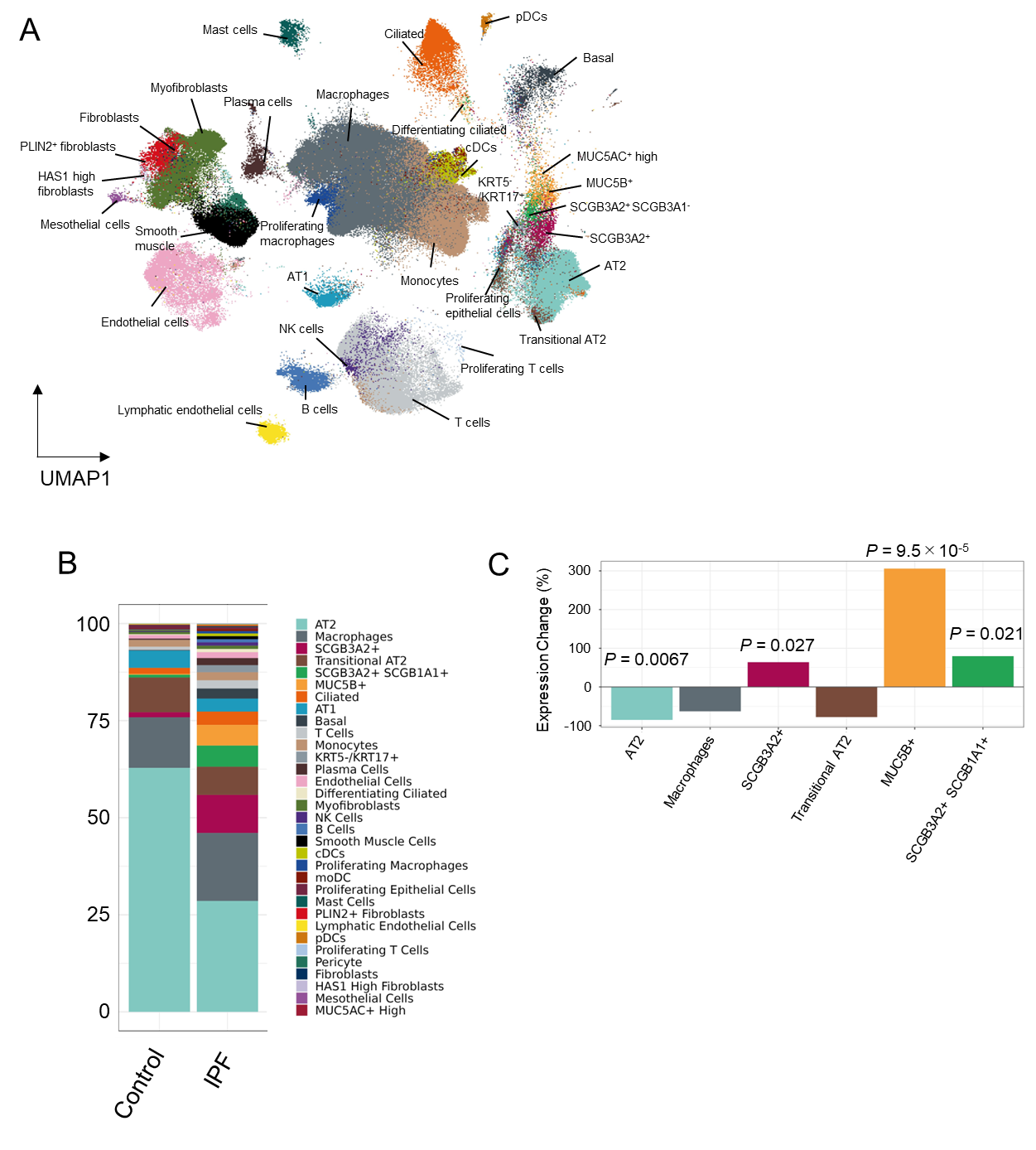


(A) Uniform Manifold Approximation and Projection embedding of jointly analysed single-cell transcriptomes from 358,047 cells from 55 IPF and 52 control lungs. (B) Bar plots show the composition of the cellular origin of SFTPB gene expression. (C) Expression changes in the differential expression analysis are shown for the six major SFTPB source cell types. The P-values with a significant association are indicated.

**Figure E10. Comparison between SFTPB and SCGB3A2 in serum EVs**


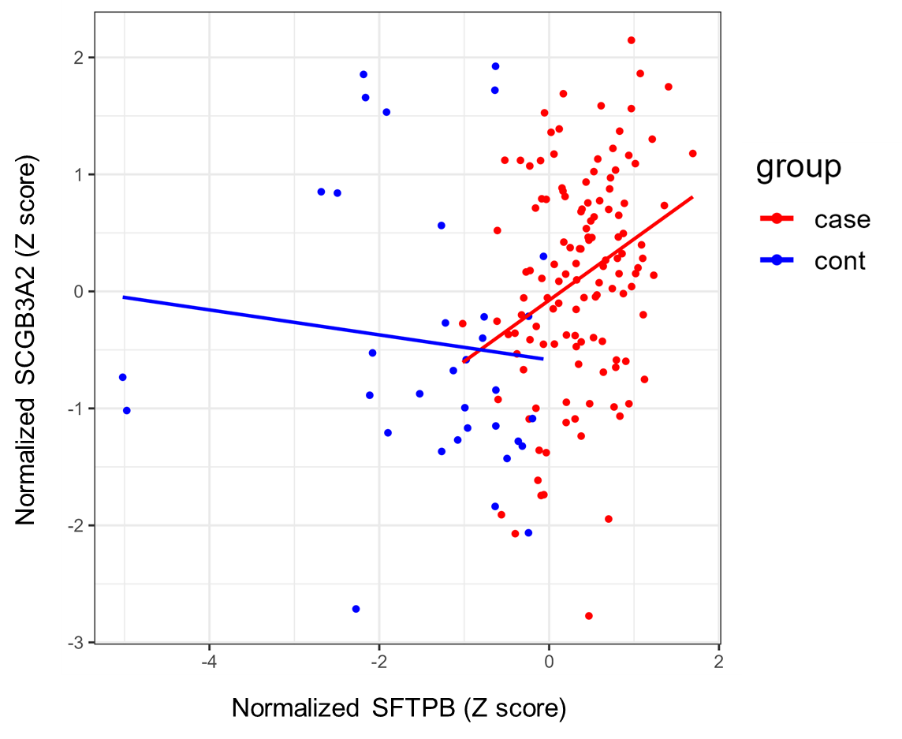


A scatter plot showing the normalized expression of SFTPB against the normalized expression of SCGB3A2 in serum EVs. The lines represent regression lines for each IPF (effect size = 0.52, *P* = 9.5×10^-4^) or control (effect size = -0.11, *P* = 0.55).

**Table E1. Summary of the study participants**

|  |  |  |
| --- | --- | --- |
| Participants for the case-control association test | | |
| Characteristics | IPF (N = 127) | Healthy control (N = 34) |
| Mean age (sd) | 72.3(9.8) | 68.9(10.0) |
| Men (%) | 96(75.6%) | 16(47.1%) |
| Smoking history |  |  |
| Current | 5(3.9%) | 0 |
| Former | 94(74.0%) | 11(32.4%) |
| Never | 21(16.5%) | 23(67.6%) |
| No data | 7(5.5%) | 0 |
| Antifibrotic therapy |  |  |
| Yes | 29(22.8%) | 0 |
| No | 98(77.2%) | 0 |
| Participants with serum KL6 values for the comparison test of  diagnostic performance and clinical features | | |
| Characteristics | IPF (N = 121) | Healthy control (N = 13) |
| Mean age (sd) | 72.6(9.6) | 75.4(6.6) |
| Men (%) | 91(75.2%) | 6(46.2%) |
| Fibrosis region on CT |  | - |
| 50%< | 23 | - |
| 10–50% | 73 | - |
| <10% | 25 | - |
| %FVC | 70.7(19.9) | - |
| Progressive^†^ | 70(57.9%) | - |
| Progressive  (post blood collection) **^‡^** | 22(18.1%) | - |
| Complication |  | - |
| Emphysema | 47(38.8%) | - |
| Lung cancer | 10(8.3%) | - |
| Participants in the replication cohort | | |
| Characteristics | IPF (N = 20) | Healthy control (N = 23) |
| Mean age (sd) | 68.6(11.5) | 69.7(12.5) |
| Men (%) | 18(90.0%) | 12(52.2%) |

Continuous data are presented as mean values (SD, standard deviation). %FVC, forced vital capacity; CT, computed tomography; IPF, idiopathic pulmonary fibrosis

^†^Progressive phenotype was defined using the criteria of the INBUILD trial.

**^‡^**Progressive phenotype that met criteria after the blood collection.

**Table E2. Diagnostic performance of the IPF-associated proteins and serum KL6**

| Markers | AUC | Specificity | Sensitivity |
| --- | --- | --- | --- |
| SFTPB | 0.97 | 1 | 0.86 |
| BPIFB1 | 0.85 | 0.85 | 0.72 |
| PPIA | 0.75 | 0.85 | 0.65 |
| KL6(serum) | 0.89 | 0.69 | 0.95 |

Specificity and sensitivity were calculated based on Youden index. AUC, area under the curve; IPF, idiopathic pulmonary fibrosis; SFTPB, surfactant protein B

**Table E3.** **Results of the association tests for the clinical features of IPF**

| Features | Protein(s) | Effect size | Standard error | *P***^†^** |
| --- | --- | --- | --- | --- |
| Fibrotic region on CT  (<10% vs control) | SFTPB | 1.54 | 0.38 | **0.00024** |
|  | BPIFB1 | 0.69 | 0.36 | 0.065 |
|  | KL6(serum) | 0.79 | 0.23 | **0.0020** |
|  | Complement module | -0.19 | 0.26 | 0.48 |
|  | SFTPA**^‡^** | 0.29 | 0.30 | 0.33 |
|  | SCGB3A1**^‡^** | 1.13 | 0.32 | **0.0011** |
|  | CCL18**^‡^** | 0.93 | 0.37 | **0.017** |
|  | POSTN**^‡^** | 0.50 | 0.36 | 0.17 |
| Fibrotic region on CT  (<10% → 50%<) | SFTPB | 0.49 | 0.13 | **0.00037** |
|  | BPIFB1 | 0.33 | 0.13 | **0.017** |
|  | KL6(serum) | 0.31 | 0.13 | **0.023** |
|  | Complement module | 0.44 | 0.13 | **0.0015** |
|  | SFTPA**^‡^** | 0.35 | 0.14 | **0.012** |
|  | SCGB3A1**^‡^** | 0.31 | 0.13 | **0.022** |
|  | CCL18**^‡^** | 0.058 | 0.14 | 0.68 |
|  | POSTN**^‡^** | 0.11 | 0.14 | 0.44 |
| %FVC | SFTPB | -0.014 | 0.0063 | **0.034** |
|  | BPIFB1 | -0.0016 | 0.0059 | 0.79 |
|  | KL6(serum) | -0.0053 | 0.0069 | 0.45 |
|  | Complement module | -0.0059 | 0.0068 | 0.39 |
|  | SFTPA**^‡^** | 0.00057 | 0.0070 | 0.93 |
|  | SCGB3A1**^‡^** | -0.012 | 0.0070 | 0.11 |
|  | CCL18**^‡^** | -0.0059 | 0.0062 | 0.35 |
|  | POSTN**^‡^** | -0.012 | 0.0061 | **0.045** |
| %DLCO | SFTPB | -0.013 | 0.0040 | **0.0015** |
|  | BPIFB1 | -0.014 | 0.0043 | **0.0023** |
|  | KL6(serum) | -0.013 | 0.0040 | **0.0024** |
|  | Complement module | -0.012 | 0.0043 | **0.0049** |
|  | SFTPA**^‡^** | -0.0099 | 0.0042 | **0.022** |
|  | SCGB3A1**^‡^** | -0.014 | 0.0040 | **0.00059** |
|  | CCL18**^‡^** | -0.0037 | 0.0044 | 0.40 |
|  | POSTN**^‡^** | -0.0055 | 0.0041 | 0.18 |
| Progressive  vs  non-progressive | SFTPB | 0.49 | 0.18 | **0.0074** |
|  | BPIFB1 | 0.12 | 0.18 | 0.53 |
|  | KL6(serum) | 0.49 | 0.18 | **0.0066** |
|  | Complement module | 0.37 | 0.18 | **0.043** |
|  | SFTPA**^‡^** | 0.43 | 0.18 | **0.018** |
|  | SCGB3A1**^‡^** | 0.019 | 0.18 | 0.92 |
|  | CCL18**^‡^** | 0.073 | 0.19 | 0.70 |
|  | POSTN**^‡^** | 0.054 | 0.18 | 0.77 |
| Progressive  (after blood collection)  vs  non-progressive | SFTPB | 0.66 | 0.27 | **0.018** |
|  | BPIFB1 | -0.06 | 0.28 | 0.84 |
|  | KL6(serum) | 0.51 | 0.24 | **0.036** |
|  | Complement module | -0.00010 | 0.25 | 1.00 |
|  | SFTPA**^‡^** | 0.50 | 0.24 | **0.045** |
|  | SCGB3A1**^‡^** | -0.035 | 0.25 | 0.89 |
|  | CCL18**^‡^** | -0.025 | 0.25 | 0.92 |
|  | POSTN**^‡^** | 0.079 | 0.25 | 0.75 |
| Emphysema | SFTPB | -0.52 | 0.19 | **0.0081** |
|  | BPIFB1 | 0.015 | 0.20 | 0.94 |
|  | KL6(serum) | 0.10 | 0.19 | 0.60 |
|  | Complement module | -0.018 | 0.20 | 0.93 |
|  | SFTPA**^‡^** | -0.13 | 0.20 | 0.52 |
|  | SCGB3A1**^‡^** | -0.33 | 0.19 | 0.089 |
|  | CCL18**^‡^** | -0.35 | 0.20 | 0.079 |
|  | POSTN**^‡^** | -0.28 | 0.20 | 0.15 |
| Lung cancer | SFTPB | -0.30 | 0.33 | 0.37 |
|  | BPIFB1 | 0.12 | 0.33 | 0.72 |
|  | KL6(serum) | 0.84 | 0.32 | **0.0095** |
|  | Complement module | 0.045 | 0.33 | 0.89 |
|  | SFTPA**^‡^** | -0.18 | 0.33 | 0.60 |
|  | SCGB3A1**^‡^** | -0.22 | 0.33 | 0.49 |
|  | CCL18**^‡^** | -0.26 | 0.33 | 0.44 |
|  | POSTN**^‡^** | -0.26 | 0.33 | 0.44 |

**^†^**Associations with *P* < 0.05 are highlighted in bold.

**^‡^**Serum exosome abundance for previously reported IPF-associated proteins.

%FVC, forced vital capacity; IPF, idiopathic pulmonary fibrosis; SFTPB, surfactant protein B; SFTPA, surfactant protein A; POSTN, periostin
